# Structures of a P4-ATPase lipid flippase in lipid bilayers

**DOI:** 10.1007/s13238-020-00712-y

**Published:** 2020-04-17

**Authors:** Yilin He, Jinkun Xu, Xiaofei Wu, Long Li

**Affiliations:** 1 State Key Laboratory of Membrane Biology, Peking-Tsinghua Center for Life Sciences, School of Life Sciences, Peking University, Beijing, 100871 China; 2 Academy for Advanced Interdisciplinary Studies, Peking University, Beijing, 100871 China

**Dear Editor,**

Phospholipid molecules are unevenly distributed in membrane bilayers of eukaryotic cells. Phosphatidylethanolamine (PE) and phosphatidylserine (PS) are concentrated in the cytoplasmic leaflet, whereas phosphatidylcholine (PC) is enriched in the exoplasmic leaflet (lumenal or extracellular leaflet) (van Meer et al. 2008). Type 4 P-type ATPases (P4-ATPases) are the phospholipid flippases which move specific lipids from the exoplasmic leaflet to the cytoplasmic leaflet. P4-ATPases belong to the P-type ATPase family. P4-ATPases are conserved in eukaryotes. *Saccharomyces cerevisiae* (*S*. *cerevisiae*) has five P4-ATPases characterized, namely Drs2p, Dnf1p, Dnf2p, Dnf3p, and Neo1p, whereas 14 P4-ATPases are identified in human. Each P4-ATPase has its preferred lipid substrates. For example, yeast Drs2p translocates PS and PE, whereas Dnf1p and Dnf2p prefer PC, PE, and glucosylceramide (Roland et al. 2019). Most P4-ATPases form heterodimers with a β-subunit from the CDC50 family. The β-subunit is required for proper folding, sub-cellular targeting, and lipid flipping of P4-ATPases (Radji et al. 2001; Bryde et al. 2010). Lipid flipping by P4-ATPases is coupled with ATP hydrolysis and enzyme phosphorylation/dephosphorylation. P4-ATPases undergo the E1–E2 state transition during lipid flipping cycles. Lipid substrate binding is coupled with dephosphorylation in the low energy E2P state whereas the role of the E1 state in lipid flipping is less clear. The lipid flipping pathway in P4-ATPases is also under debate. A “two-gate” model suggests that specific phospholipids are recognized at the entry and exit gates by the residues clustering at the exoplasmic and cytoplasmic ends of transmembrane segments (TMs) 1–4. The polar heads of the lipid substrates could slide through a groove formed by TMs 1, 3, and 4 during flipping (Baldridge and Graham 2013). A “hydrophobic gate” model proposed a cross-membrane groove bordered by TMs 1, 2, 4, and 6. A highly conserved isoleucine residue (I364 in bovine ATP8A2) and a few hydrophobic residues nearby act as a hydrophobic gate to control lipid movements (Vestergaard et al. 2014). A third model proposed that a central cavity between TMs 3, 5, and 6 could accommodate the head groups of phospholipids during transporting across the membranes (Jensen et al. 2017).

Recently, the electron cryo-microscopy (cryo-EM) structures of *S. cerevisiae* Drs2p-Cdc50p (scDrs2p-Cdc50p) (Timcenko et al. 2019) and human ATP8A1-CDC50a (hATP8A1-CDC50a) (Hiraizumi et al. 2019) solubilized in detergents were reported. The structures of scDrs2p-Cdc50p were determined in the E2P state and the hATP8A1-CDC50a structures in several E1 and E2 intermediate states. However, the subtle movements of TMs among the structures make it hard to draw a consensus model for lipid flipping. Here, we report the cryo-EM structures of a P4-ATPase from a thermophilic fungus reconstituted into lipid nanodiscs. The structures are determined in the presence of β, γ-methyleneadenosine 5′-triphosphate (AMPPCP) and beryllium fluoride (BeF_3_^−^), representing the E1-ATP and E2P states, respectively. The large conformational changes between the two states suggest a mechanism underlying lipid flipping by P4-ATPases during the E1–E2 transition.

Dnf1p and Cdc50p from a thermophilic fungus, *Chaetomium thermophilum* (*C*. *thermophilum*) were cloned for structural studies (ctDnf1p-Cdc50p) (Fig. S1). The purified ctDnf1p-Cdc50p heterodimer showed phospholipid-dependent ATPase activity (Fig. S2). Both phosphatidylcholine (PC) and phosphatidylserine (PS) could stimulate ATPase activity, with PC slightly better than PS at high concentrations (Fig. S2d). In contrast, scDrs2p was shown to be selectively stimulated by PS (Zhou and Graham 2009). Therefore, ctDnf1p is likely to have different substrate preference from scDrs2p, maybe preferring PC to PS, similar to scDnf1p and scDnf2p (Baldridge and Graham 2013). To mimic the native lipid membrane environment, ctDnf1p-Cdc50p was reconstituted into lipid nanodiscs (Bayburt et al. 2002). The complex was supplemented with AMPPCP (E1-ATP) or BeF_3_^−^ (E2P) and subject to cryo-EM single-particle analyses. The structures were determined to a resolution of 3.5 Å for E1-ATP and of 3.4 Å for E2P (Figs. S3–5).

Similar to scDrs2p and hATP8A1, ctDnf1p has all the typical domains of P-type ATPases, namely the actuator domain (A), the nucleotide-binding domain (N), the phosphorylation domain (P), and the membrane domain (M) (Fig. 1A and 1B). Comparison of the ctDnf1p-Cdc50p structures in the E1-ATP and the E2P states shows that ctCdc50p and TMs 3–10 of ctDnf1p are quite similar (root mean square deviation = 0.79 Å), whereas the largest differences are seen in the N domain, the A domain, and TMs 1-2 of ctDnf1p (Fig. S6). In the E2P structure, BeF_3_^−^ is bound at the phosphorylation site D606 (Fig. S5h), same as in the typical E2P structures of P-type ATPases (Timcenko et al. 2019; Hiraizumi et al. 2019). The A domain associates tightly with the N and P domains. Both A and N domains are in an upright conformation (Fig. 1A and 1B). In the E1-ATP structure, AMPPCP adapts an extended conformation (Fig. S5i), different from the bent conformations observed in hATP8A1 and other P-type ATPases (Fig. S7) (Hiraizumi et al. 2019). The extended conformation suggests the structure may represent a distinct active E1-ATP state not observed before (Lu et al. 2014). As a result, the N domain rotates by 35° compared to its orientation in the E2P structure (Fig. S6c). The A domain has even larger conformational changes. It moves close to the M domain, no longer associated with the N and P domains (Fig. 1B). The displacement of A domain is about 30 Å (Fig. S6b), the largest among the known structural changes of P-type ATPases during the E2–E1 transition. TMs 1 and 2 that are directly connected to the A domain have high flexibility in the E1-ATP state. The entire TM1 including the amphipathic helix (residues 122–134) and L1/2 are invisible in the density map, indicating its high flexibility. The C-terminal segment of TM2 that extend to domain A (residues 185–192) is bent and largely disordered. The TM2 density in E1-ATP is also weaker than in E2P, suggesting a more flexible TM2 in E1-ATP. Indeed, in the refined models, the average B factors of TM2 (residues 158–184) are 38.5 in E2P and 71.7 in E1-ATP.

**Figure 1:**
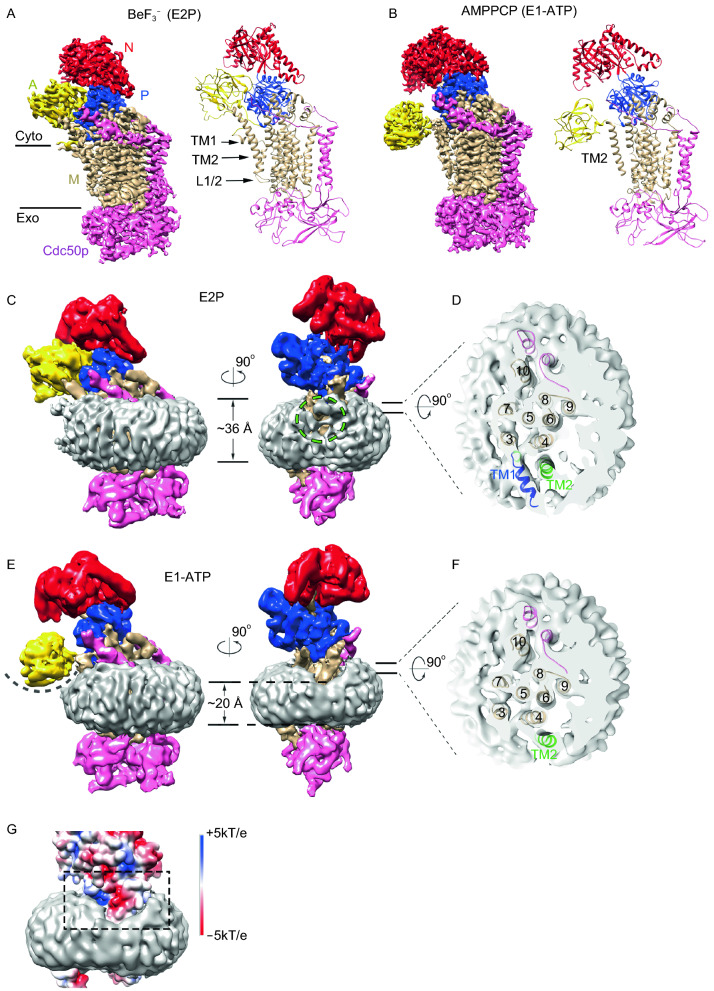
**Structures of ctDnf1p-Cdc50p**. (A) Cryo-EM map (left panel) and ribbon diagram (right panel) of ctDnf1p-Cdc50p with BeF_3_^−^. The A, N, P, and M domains and Cdc50p are labeled and colored yellow, red, blue, tan, and pink, respectively. (B) Cryo-EM map (left panel) and ribbon diagram (right panel) of ctDnf1p-Cdc50p with AMPPCP. Colors are the same as in (A). (C) Cryo-EM density map of the E2P state with the lipid nanodisc. The map is low pass filtered to 6 Å. Colors are as in (A) with the addition of lipid nanodisc density (grey). Two views differing by 90° are shown. The green dashed circle marks the nanodisc density around TMs 1 and 2. The A domain is omitted in the right panel for clarity. (D) Cut-away top view of the nanodisc in (C) (right panel). TMs are labeled. (E) Cryo-EM density map of the E1-ATP state with the lipid nanodisc. The curved dashed line indicates the depression that is required to fit the A domain on the surface of the membranes. Membrane thickness is labeled. (F) Cut-away top view of the nanodisc in (E) (right panel). (G) Zoom-in view of (E) (right panel). The surface of ctDnf1p is colored according to the electrostatic potential. The black dashed frame highlights the distorted membranes and the negatively charged patch

The structures of ctDnf1p-Cdc50p in lipid nanodiscs not only allow us to visualize conformational changes of the flippase during the E1–E2 transition, but also to analyze the structural changes of the membranes. The cryo-EM density maps clearly show that the M domain is wrapped in lipid nanodiscs. In the E2P structure (Fig. 1C), the amphipathic helix of TM1 lies on the surface of the lipid membranes, extending to the brink of the nanodisc (Fig. 1D). The side wall of the nanodisc close to TMs 1 and 2 is not well sealed, indicating that the local lipid bilayers might be disturbed (Fig. 1C). Nevertheless, the nanodisc has a roughly even thickness of ~36 Å. In the E1-ATP structure (Fig. 1E and 1F), the lipid bilayers close to TMs 1 and 2 are dramatically distorted. The local membranes shrink to ~20 Å in thickness. Lipid thinning exposes a negatively charged segment of TM2 (residues 174–181) that is shielded away from the lipids in the E2P structure by interacting with the amphipathic helix of TM1. In the context of the native membranes, lipid thinning would result in a depression on the membrane surface to fit the A domain (Fig. 1E). The structure suggests that the large displacement of the A domain in the E1-ATP state might dislodge TM1, which becomes highly flexible and expose a negatively charged patch (Fig. 1G). The patch forming residues, D175, E178 of TM1 and E561 of TM4 are highly conserved among P4-ATPases (Fig. S1). The flexible TM1 and the charged patch might have the ability to distort the local membranes whose thickness is shrunk by almost a half (Fig. 1G). The A domain on the membrane surface may help to distort the local bilayers as well. Interestingly, local membrane thinning and distortion have also been observed for the lipid scramblase TMEM16F (also known as ANO6) (Feng et al. 2019). It should be noted that membrane thinning and distortion are seen at the edge of the nanodisc where the membrane scaffold protein (MSP) wraps around the lipids. Although the MSP could not be resolved in the density maps, it may be involved during local membrane changes as well. Further studies are required to clarify the issue.

Distinct lipid binding sites are identified in the E2P and E1-ATP structures of ctDnf1p-Cdc50p. In the E2P structure, a phospholipid molecule is found in a cavity formed by TMs 2, 4, and 6 in the exoplasmic half of the M domain (E2-site1) (Figs. 2A, 2B and S8a). A prominent density blob corresponding to a phospholipid snorkels deep into the positively charged cavity. A PC molecule is modeled in the density, though it could be other kinds of phospholipids as the resolution is insufficient to distinguish the head groups. The head group is surrounded by residues Q549 and N550 of TM4 and N1153 of TM6 (Fig. 2A). The following acyl chains bent almost 90°, extending into the membranes. A similar lipid binding site is observed in the E2Pi structure of hATP8A1 (AlF_4_^−^ bound) (Hiraizumi et al. 2019) (Fig. S8b). In that structure, the serine group of the modeled PS molecule interacts with N352 of hATP8A1 (equivalent to Q549 in ctDnf1p), whereas the head group density in our structure points deeper into the cavity (Fig. S8c). In addition, the acyl chains of PS run along hATP8A1 pointing to the cytosolic side, unlike the acyl chains that go into the middle of the lipid bilayers in our structure. Therefore, the E2-site1 is likely for early recognition of the phospholipid substrates that have been picked up by the flippase, but are still located in the exoplasmic leaflet. A second lipid molecule is identified at the cytoplasmic end of the groove formed by TMs 2, 4, and 6 (E2-site2) (Fig. 2A and 2B). The phosphate head group is at the water-membrane interface, clamped by R181 of TM2, F569 and Y572 of TM4, which are not conserved among P4-ATPase though. The acyl chains run along the groove, with the same orientation as the lipids in the cytoplasmic leaflet. Therefore, it might represent the phospholipid that has finished flipping and is ready to be released to the cytoplasmic leaflet. It remains to be seen whether the site is conserved among P4-ATPases.

**Figure 2:**
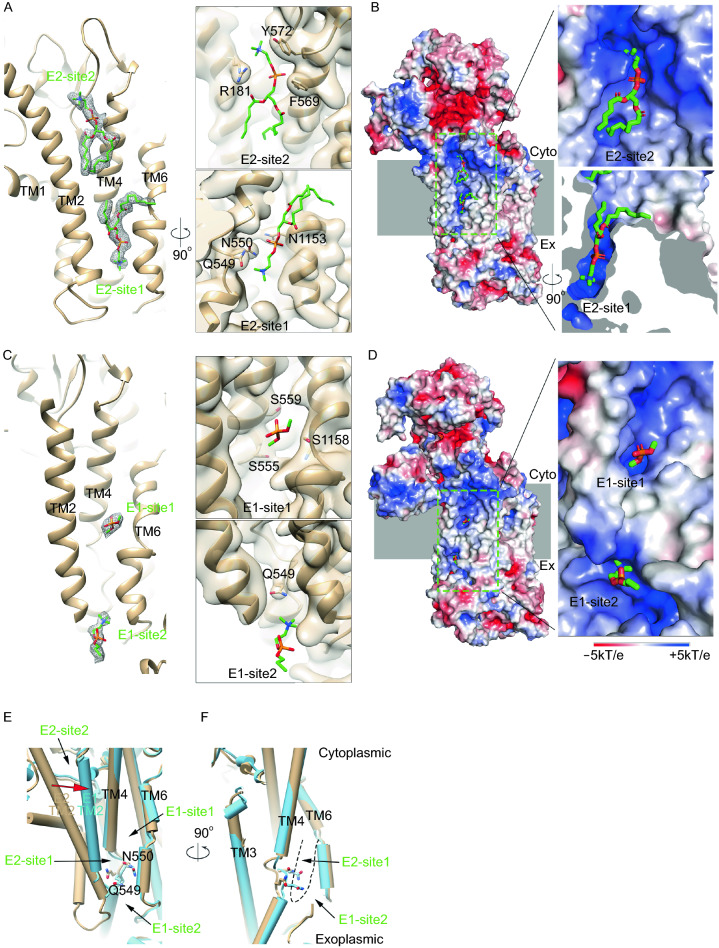
**Lipid substrate binding sites**. (A) Lipid binding sites in E2P. ctDnf1p is shown as ribbon. The density of the lipids is shown as grey meshes at 1.5σ. Two modeled PC and their interacting residues are shown as sticks. The left panel shows the overview and the right panels show the magnified views of each site. TMs, residues, and binding sites are labeled. (B) Electrostatic potential surfaces of the flippase in E2P, showing the environment of the lipid binding sites. The electrostatic potential surfaces are calculated using APBS with the default setting in PyMOL. The membrane cartoon is colored grey. The left panel shows the overview and the right panels show the magnified views of each site. (C and D) Same as (A) and (B) except showing the binding sites in the E1-ATP structure. A phosphate head group and a lyso-PC are modelled in site 1 and 2, respectively. (E) Superimposition of E1-ATP (cyan cylinders) and E2P (tan cylinders), showing the groove bordered by TMs 2, 4, and 6. Q549 and N550 that interact with the lipid substrate are shown as sticks. The movement of TM2 during the E2–E1 transition is indicated by a red arrow. The lipid binding sites are marked. (F) Side view of the groove. The dashed line outlines E2-site1 which is disrupted and occupied by Q549 and N550 in E1-ATP

In the E1-ATP structure, a relatively small but distinct density blob is found halfway through the membranes in the groove bordered by TMs 2, 4, and 6 (E1-site1) (Fig. 2C and 2D), two helices away from E2-site1. The density could be the head group of a phospholipid or a hydrophilic molecule as it is close to S1158 of TM6 and points to highly conserved S555 and S559 of TM4 in the groove (Fig. S1). The elongated density blobs are also found at the same locations in the scDrs2p and hATP8A1 structures (Fig. S9), though they are not discussed in the papers (Timcenko et al. 2019; Hiraizumi et al. 2019). This spot may serve as a transient yet conserved binding site in the middle of the phospholipid flipping pathway. A mutation in ATP8B1, S403Y (equivalent to S555 in ctDnf1p), was found in the patients with progressive familial intrahepatic cholestasis type 1 (PFIC1) (Klomp et al. 2004), suggesting an important role of the site in lipid flipping.

The distorted lipid bilayers and the disordered TM1 and L1/2 in the E1-ATP structure pose an opportunity for phospholipids to enter the groove between TMs 2, 4, and 6. Indeed, a rod-like density blob is found at the opening between the exoplasmic ends of TMs 2 and 6, on the surface of the membranes (Fig. 2C and 2D) (E1-site2). The side chains of Q549 and N550 of TM4 in the groove are in proximity to the density, in a position ready to pick a phospholipid from the membranes. Due to the low local resolution, it is not entirely certain whether the density blob represents a phospholipid that is entering the groove. Nevertheless, the wide opening between TMs 2 and 6 and the local positive charges (Fig. 2D) suggest it is a promising lipid entry site.

The difference in lipid binding between the E1-ATP and E2P structures is mainly caused by the conformational changes of TM2 and TM4. As E2P shifts to E1-ATP, E2-site1 becomes too small to accommodate the head group of a phospholipid (Fig. 2E and 2F). The local main chain changes of TM4 move the side chains of Q549 and N550 towards the membranes, occupying the space that is filled by the head group of the phospholipid in the E2P structure (Fig. 2F). The shift of TM2 towards TM4 further narrows the path that allows the acyl chains of the lipid to come out from the cavity, while creates a shallow lipid binding site, E1-site1. The disordered L1/2 and the exoplasmic end of TM2 in the E1-ATP structure would allow the phospholipids to enter from E1-site2 (Fig. 2E), where the “cavity-occupying” Q549 and N550 have access to the phospholipids through the opening between TMs 2 and 6 (Fig. 2C and 2E). On the cytoplasmic side, the large shift of TM2 towards TM4 disrupts E2-site2, leaving no space for lipid binding (Fig. 2E).

In summary, the structures of ctDnf1p-Cdc50p suggest that P4-ATPases have evolved a unique mechanism for lipid flipping. TMs 3–10 of P4-ATPases are kept in a relatively fixed conformation by Cdc50p during ATP hydrolysis. TMs 1 and 2 are the major regulators for lipid flipping. The distorted lipid bilayers and the groove bordered by TMs 2, 4, and 6 may be the key factors in controlling lipid flipping. It remains to be seen how the P4-ATPases select specific lipids to enter and exit the flipping pathway.

## Electronic supplementary material

Below is the link to the electronic supplementary material.

Supplementary material 1 (PDF 7282 kb)
