# Supplementary material for: Structures of a P4-ATPase lipid flippase in lipid bilayers
Source: Protein Cell. 2020 Apr 17;11(6):458–63. doi: 10.1007/s13238-020-00712-y (PMC7251018; doi:10.1007/s13238-020-00712-y)

Supplementary Information

Structures of a P4-ATPase lipid flippase in lipid bilayers

Yilin He[1,2], Jinkun Xu[1], Xiaofei Wu[1], Long Li[1]#

[1] State Key Laboratory of Membrane Biology, Peking-Tsinghua Center for Life Sciences, School of Life Sciences, Peking University, Beijing, China

[2] Academy for Advanced Interdisciplinary Studies, Peking University, Beijing, China

# Corresponding author: Long Li; email: long_li@pku.edu.cn

Contents

Supplemental results and discussion

Materials and methods

Supplementary Figures 1-13

Supplementary Table 1

Supplemental results and discussion

Results

In both E1-ATP and E2P structures, ctCdc50p has extensive interactions with ctDnf1p. On the cytoplasmic side, the N-terminal peptide (residues 23-46) of ctCdc50p runs along one face of ctDnf1p, interacting with the cytosolic loops of ctDnf1p, including the segment connecting TM4 and the P domain, the loop between TMs 6 and 7 (L6/7), L8/9, and a C-terminal amphipathic helix that was suggested to undergo conformational changes upon PI4P activation in scDrs2p[1,2] (Fig. S10). The C-terminal tail of ctCdc50p (residues 384-398), which is invisible in the scCdc50p and hCDC50a structures, could be traced to run towards ctDnf1p. Thus, the C-terminal tail and the N-terminal peptide of ctCdc50p are in such a conformation that sandwiches the C-terminal amphipathic helix of ctDnf1p (Fig. S10). The two TMs of ctCdc50p form hydrophobic interactions with TM7 and TM10 of ctDnf1p. The ectodomain of ctCdc50p has the largest interfaces with ctDnf1p, interacting with all the exoplasmic loops except L1/2 (Fig. 1a). Thus, ctCdc50p acts as a 3-way clamp to hold TMs 3-10 of ctDnf1p in a relatively fixed conformation in both E1-ATP and E2P structures.

The E1-ATP and E2P structures have a lipid-binding site in common in a cavity formed by TMs 7, 8, and 10, on the opposite side of TMs 2, 4, and 6 (Fig. S11). Phospholipid density is observed at the cytoplasmic border of the membranes. The cavity was suggested to be a PI4P binding site for scDrs2p activation[1]. However, in our structures, the head group does not insert as deep as PI4P in scDrs2p $E2P^{inter}$ or $E2P^{active}$. Instead, it is likely to be the head group of PS as modeled in scDrs2p $E2P^{inhib}$ [1].

Discussion

Phospholipid flipping coupled with the E1-E2 transition

Four lipid-binding sites are identified in the groove formed by TMs 2, 4, and 6, two from E1-ATP and two from E2P. The four sites arranged in such a way that they could relay the phospholipid substrates through the groove during the E1-E2 transition (Fig. S12). Conformational changes of TM1 and TM2 guide the phospholipids to move from the exoplasmic leaflet to the cytoplasmic leaflet. ATP binding to the lipid flippase in the E1-ATP state detaches the A domain from the N and P domains. The large motion of the A domain increases the flexibility of TMs 1 and 2 and exposes a negatively charged patch formed by the residues from TMs 2 and 4. The local lipid bilayers are distorted and the membranes are thinned by almost a half. In the distorted membranes, the phospholipid molecules are more likely to tilt parallel to the

membrane plane. The lipid head groups are in an inward-facing orientation, ready to enter the groove via E1-site2 (Fig. S12a). In the E2P state, the A domain is associated with the P and N domains tightly, leading to a relatively rigid conformation of TMs 1 and 2. The local lipid bilayer structures are restored. TMs 2, 4, and 6 create a cavity (E2-site1) in the exoplasmic leaflet for shielding the polar head group of the phospholipid that has been picked up from E1-site2 (Fig. S12b). E2-site1 is disrupted in the E1-ATP state as TM2 moves towards TMs 4 and 6 and two polar residues of TM4 (Q549 and N550) lean towards the membranes. The lipid head group is likely to be squeezed out and to move forward to the cytoplasmic leaflet via E1-site1, a shallow hydrophilic cleft (Fig. S12c). Finally, the phospholipid reaches E2-site2 and is held by a clamp between TMs 2 and 4 in a flipped conformation (Fig. S12d). The phospholipid substrate is ready to be laterally released into the cytoplasmic leaflet when the clamp is disrupted in the E1-ATP state. In the scenario proposed above, it takes two E1-E2 cycles to flip one phospholipid substrate, but costs one ATP molecule per phospholipid on average because two phospholipids are present at the same time in each state. However, as we are missing several intermediate states, e.g. E2 and E1P, the exact cycle number and ATP cost per substrate need further investigation. During lipid transport, the hydrophilic head group of the phospholipid substrate is constantly protected from the hydrophobic environment by sliding through the binding sites in the positively charged groove (Fig. 2b, d). The groove provides the only continuous hydrophilic pathway in the M domain during the E1-E2 transition (Compare Fig. 2b, d and Fig. S11c). The positive charges are mainly contributed by K174, R181, and K1121 on the TMs. The highly conserved K1121 has been shown to be important for substrate binding and ATPase activity[3]. Consistent with biochemical data[3], the groove has high affinity to the lipid substrate in the E2P state as evident by the strong phospholipid density, whereas the groove shows weak lipid binding to facilitate lipid entry and exit in the E1-ATP state as indicated by the fragmented lipid density in the structure. Similarly, a hydrophilic membrane-traversing groove is also present in the TMEM16F scramblase[4].

The “hydrophobic gate” model suggests that TMs 1 and 2 move away from TMs 3 and 4 during lipid transport[5]. Indeed, our structures show that TM1 and TM2 becomes flexible in E1-ATP. The key residue, I364 of the “hydrophobic gate” (I554 in ctDnf1p), is at the interface between TMs 1, 2, and 4 (Fig. S13). Thus the mutations of the residue may disrupt the E1-E2 equilibrium and hamper lipid flipping as observed in the mutagenesis studies[5]. The “two-gate” model suggests that the flippases recognize the phospholipid substrates by interacting with the head groups. Residues other than the classical ion binding residues in ion-pumping P-type ATPases are involved in recognition[6-9]. Consistent with the model, the four distinct binding sites in our structures mainly interact with the lipid head groups. However, the sites do not seem to provide a discrimination mechanism for different phospholipid substrates. As shown in the phospholipid-dependent ATPase activity assays, ctDnf1p may have different

substrate specificity from scDrs2p and hATP8A1. The amino acids that interact with the polar head group of the phospholipid substrate at E2-site1 are similar to those in the structures of scDrs2p and hATP8A1. The corresponding residues are Q549 and N550 of ctDnf1p, S503 and N504 of scDrs2p, and N352 and N353 of hATP8A1 (Fig. S1). The clamp residues of E2-site2 consist of both hydrophilic and hydrophobic residues, but are not conserved among P4-ATPases (Fig. S1). The serine residues at E1-site1 are highly conserved among P4-ATPases, and E1-site2 only provides a steric opening. Further studies on other intermediate states may provide clues on the substrate specificity.

Materials and methods

Protein expression and purification

Protein BLAST search identified three P4-ATPases in *C. thermophilum*, including one *S. cerevisiae* Drs2p homolog (ctDrs2p), one Dnf1p and Dnf2p homolog (ctDnf1p), and one Dnf3p homolog (ctDnf3p) (Fig. S1). Only one CDC50 protein was found in the *C. thermophilum* genome (ctCdc50p). After initial screening, ctDnf1p was chosen to co-express with ctCdc50p in yeast. The genes of ctDnf1p and ctCdc50p were cloned from the cDNA library of *Chaetomium thermophilum* (*var. thermophilum* strain: DSM1495, a gift from Dr. Stefan Schoebel). Superfolder green fluorescence protein (sfGFP)[10], a Twin-Strep tag and a 3C protease cleavage site were fused to the N-terminus of ctDnf1p. sfGFP, a His_9 tag, and a 3C protease cleavage site were fused to the N-terminus of ctCdc50p. The expression plasmids pRS426-sfGFP-twinStrep-3C-ct*DNF1* and pRS424-sfGFP-His_9-3C-ct*CDC50* were co-transformed into *S. cerevisiae* strain BJ5465 using the LiAc/SS carrier DNA/PEG method[11]. Yeast cells were cultured in synthetic drop-out medium supplemented with 2% raffinose at 30 °C for about 24h to reach an optical density (OD_{600}) of about 5. The culture was induced by the addition of 2% galactose and continued for 20 h at 25°C. The cells were harvested and stored at −80 °C until use.

The cells were suspended in the membrane extraction buffer (20 mM Tris-HCl pH 7.4, 150 mM NaCl, 5mM $MgCl_2$,1mM DTT, and protease inhibitor cocktails) and lysed by high pressure homogenization. The crude lysate was clarified by centrifugation (20,000×g, 25 min, 4°C). The membrane fraction was pelleted by ultracentrifugation (200,000×g, 1 h, 4°C) and washed once with the membrane extraction buffer. The membrane pellets were solubilized in 2% lauryl maltose neopentyl glycol (LMNG, Anatrace) in the membrane solubilization buffer (20 mM Tris-HCl pH 7.4, 150 mM NaCl, 5mM $MgCl_2$,1mM DTT, 10% glycerol, and protease inhibitor cocktails). After incubation at 4 °C for 1 h, the solution was clarified by ultracentrifugation (200,000×g, 1 h, 4°C). The supernatant was mixed with avidin (Sigma) and loaded onto a column pre-packed with StrepTactin resin (IBA

Lifesciences). The eluents were concentrated and incubated with 3C protease at 4°C overnight. The protein solution was then loaded onto a Superdex 200 10/300 column (GE Healthcare). The peak fractions were pooled and concentrated (Fig. S2). The purified protein was either reconstituted into nanodiscs or flash-frozen in liquid nitrogen and stored at −80 °C.

The purified protein was mixed with MSP1D1[12] and yeast polar lipids (Avanti Lipids, 40 mg/ml dissolved in 1% DDM) at a molar ratio of 1:2:25. Bio-beads SM2 (Bio-Rad) were then added to the mixture and incubated at 4 °C overnight to remove detergents. The complex was further purified by size-exclusion chromatography on a Superdex 200 10/300 column. The peak fraction had a protein concentration of 1.0 mg/ml (Fig. S2). It was immediately used for cryo-EM sample preparation without concentrating.

Cryo-EM sample preparation and data collection

The freshly prepared samples were incubated with 1mM BeF_3 or 1mM AMPPCP on ice for 30min before vitrification. The cryo-grid preparation was performed at 4 °C and 100% humidity in an FEI Vitrobot Mark IV. 4 μl sample was applied to each freshly glow-discharged grid (Quantifoil, R1.2/1.3). The grids were then plunge-frozen in liquid ethane. The cryo-grids were screened with a 200 kV FEI Talos Arctica microscope equipped with a FEI Ceta camera. The data were collected on a 300 kV FEI Titan Krios TEM with a K2 summit camera and GIF Quantum energy filter (Gatan). The images were collected at a magnification of 130,000× with a calibrated pixel size of 1.055 Å. The dose rate was set at 8 e^-/s/pixel and the exposure time was 8 s, corresponding to a total dose of 57.5 $e^-/Å^2$. Movie stacks (32 frames each) were recorded with the software SerialEM[13] under low-dose conditions with defocuses ranging from -1 to -2 μm.

Image processing

The movie stacks were subject to motion correction and electron-dose weighting by using MotionCor2[14] (Fig. S3a, S4a). The program Gctf[15] was used to estimate the contrast transfer function (CTF) parameters. Images of high quality were selected for further image processing on the basis of the CTF power spectra of the corrected images. The following calculations are performed with RELION3.0[16]. Particles of high quality were selected according to 2D classification (Fig. S3b, S4b) and 3D classification results. The selected particles were subject to several rounds of CTF refinement and polishing. After mask-based post-processing, the final maps had resolutions of 3.40 Å and 3.48 Å for the AMPPCP and BeF_3^- samples, respectively (Fig. S3, S4). All the resolution estimations were based on gold-standard Fourier Shell Correlation (FSC) 0.143 criteria.

The model for the E2P (BeF_3^-) structure was built manually in Coot[17], with the guidance of the scDrs2p structures. The model was refined in real space using Phenix[18]. For the model building of E1-ATP (AMPPCP), the E2P model was fit in the E1-ATP density map. Each domain is subject to rigid body refinement. Due to the local resolution limits, the A and N domains were not refined further. The rest parts of the E1-ATP model were refined in real space with Phenix. Model validation was done with MolProbity[19]. The cryo-EM maps have been deposited in the Electron Microscopy Data Bank under accession numbers 0872 (BeF_3^-) and 0873 (AMPPCP). The atomic structure coordinates have been deposited in the Protein Data Bank under the accession number 6LCP (BeF_3^-) and 6LCR (AMPPCP). All other data can be obtained from the corresponding author upon reasonable request.

ATPase activity assay

The ATPase activity assays were carried out by using BIOMOL® Green (Enzo) to measure the free phosphate concentrations. The reaction solutions consisted of 0.05mg/ml protein, 0.01% LMNG, 0.02% $C_{12}E_9$ (Anatrace), 150 mM NaCl, 20 mM HEPES-NaOH pH 7.5, 5mM $MgCl_2$, 1mM DTT, 2.5mM ATP, and lipids at the indicated concentrations. The reactions were carried out at 30 °C for 20 min, and then immediately diluted 10 times for color development. 100 µl reagent was added to 50 µl sample and the mixture was incubated at room temperature for 20 min. The absorbance at 650 nm was measured in a microplate reader (BioTek Cytation5). The phosphate concentration was determined by calibration with the phosphate standard (BML-KI102).

References

1 Timcenko, M. *et al.* Structure and autoregulation of a P4-ATPase lipid flippase. *Nature* **571**, 366-+, doi:10.1038/s41586-019-1344-7 (2019).

2 Bai, L. *et al.* Autoinhibition and activation mechanisms of the eukaryotic lipid flippase Drs2p-Cdc50p. *Nat Commun* **10**, 4142, doi:10.1038/s41467-019-12191-9 (2019).

3 Coleman, J. A., Vestergaard, A. L., Molday, R. S., Vilsen, B. & Andersen, J. P. Critical role of a transmembrane lysine in aminophospholipid transport by mammalian photoreceptor P4-ATPase ATP8A2. *Proc Natl Acad Sci U S A* **109**, 1449-1454, doi:10.1073/pnas.1108862109 (2012).

4 Brunner, J. D., Lim, N. K., Schenck, S., Duerst, A. & Dutzler, R. X-ray structure of a calcium-activated TMEM16 lipid scramblase. *Nature* **516**, 207-212, doi:10.1038/nature13984 (2014).

5 Vestergaard, A. L. *et al.* Critical roles of isoleucine-364 and adjacent residues in a hydrophobic gate control of phospholipid transport by the mammalian P4-ATPase ATP8A2. *Proc Natl Acad Sci U S A* **111**, E1334-1343, doi:10.1073/pnas.1321165111 (2014).

6 Baldridge, R. D. & Graham, T. R. Identification of residues defining phospholipid flippase substrate specificity of type IV P-type ATPases. *Proc Natl Acad Sci U S A* **109**, E290-298, doi:10.1073/pnas.1115725109 (2012).

7 Baldridge, R. D. & Graham, T. R. Two-gate mechanism for phospholipid selection and transport by type IV P-type ATPases. *Proc Natl Acad Sci U S A* **110**, E358-367, doi:10.1073/pnas.1216948110 (2013).
8 Roland, B. P. & Graham, T. R. Directed evolution of a sphingomyelin flippase reveals mechanism of substrate backbone discrimination by a P4-ATPase. *Proc Natl Acad Sci U S A* **113**, E4460-4466, doi:10.1073/pnas.1525730113 (2016).
9 Roland, B. P. *et al.* Yeast and human P4-ATPases transport glycosphingolipids using conserved structural motifs. *J Biol Chem* **294**, 1794-1806, doi:10.1074/jbc.RA118.005876 (2019).
10 Pedelacq, J. D., Cabantous, S., Tran, T., Terwilliger, T. C. & Waldo, G. S. Engineering and characterization of a superfolder green fluorescent protein. *Nat Biotechnol* **24**, 79-88, doi:10.1038/nbt1172 (2006).
11 Gietz, R. D. & Schiestl, R. H. High-efficiency yeast transformation using the LiAc/SS carrier DNA/PEG method. *Nat Protoc* **2**, 31-34, doi:10.1038/nprot.2007.13 (2007).
12 Ritchie, T. K. *et al.* Chapter 11 - Reconstitution of membrane proteins in phospholipid bilayer nanodiscs. *Methods Enzymol* **464**, 211-231, doi:10.1016/S0076-6879(09)64011-8 (2009).
13 Mastronarde, D. N. Automated electron microscope tomography using robust prediction of specimen movements. *J Struct Biol* **152**, 36-51, doi:10.1016/j.jsb.2005.07.007 (2005).
14 Zheng, S. Q. *et al.* MotionCor2: anisotropic correction of beam-induced motion for improved cryo-electron microscopy. *Nat Methods* **14**, 331-332, doi:10.1038/nmeth.4193 (2017).
15 Zhang, K. Gctf: Real-time CTF determination and correction. *J Struct Biol* **193**, 1-12, doi:10.1016/j.jsb.2015.11.003 (2016).
16 Zivanov, J. *et al.* New tools for automated high-resolution cryo-EM structure determination in RELION-3. *Elife* **7**, doi:10.7554/eLife.42166 (2018).
17 Emsley, P., Lohkamp, B., Scott, W. G. & Cowtan, K. Features and development of Coot. *Acta Crystallogr D Biol Crystallogr* **66**, 486-501, doi:10.1107/S0907444910007493 (2010).
18 Afonine, P. V. *et al.* Real-space refinement in PHENIX for cryo-EM and crystallography. *Acta Crystallogr D Struct Biol* **74**, 531-544, doi:10.1107/S2059798318006551 (2018).
19 Chen, V. B. *et al.* MolProbity: all-atom structure validation for macromolecular crystallography. *Acta Crystallogr D Biol Crystallogr* **66**, 12-21, doi:10.1107/S0907444909042073 (2010).

Supplementary Figures

Fig. S1 Sequence alignment of selected P4-ATPases.

Sequence alignment of ctDnf1p, ctDrs2p and other P4-ATPases in yeast, bovine, human, and *A. thaliana*, aligned by T-coffee[48]. The conserved domains and transmembrane helices of ctDnf1p are indicated above the sequences. The conserved residues are indicated in red letters. The amphipathic helix of TM1 is highlighted with a green bar above the alignment. The phosphorylation site of the P domain is highlighted with a green dot. The residues involved in the negatively charged patch are highlighted with red dots. The residues contribute to the positive charge of the groove are highlighted with blue dots. The residues involved in E1-site1 are highlighted with orange dots. The residues involved in E2-site1 are highlighted with magenta dots. The residues involved in E1-site2 are highlighted with purple dots. The key isoleucine residue in the hydrophobic gate model is highlighted with a grey dot. Ct, *Chaetomium thermophilum*; Sc, *Saccharomyces cerevisiae*; Bt, *Bos Taurus*; Hs, *Homo sapiens*; At, *Arabidopsis thaliana*. Uniprot accession numbers: ScDRS2, P39524; ScDNF1, P32660; ScDNF2, Q12675; BtATP8A2, C7EXK4; HsATP8A1, Q9Y2Q0; HsATP8A2, Q9NTI2; HsATP11A, P98196; HsATP11C, Q8NB49; HsATP8B1, O43520; HsATP8B2, P98198; HsATP10A, O60312; AtALA2, P98205; AtALA10, Q9LI83.

Fig. S2 Purification and characterization of the ctDnf1p-Cdc50p complex.

a, Flow chart of ctDnf1p-Cdc50p purification. **b,** Size-exclusion chromatography profile of the protein complex reconstituted into nanodiscs. The gray-shaded area (Fraction 27) was used for cryo-EM analysis. **c,** SDS-PAGE analysis of fractions from the SEC purification in **b**. **d,** ATPase activity of ctDnf1p-Cdc50p complex stimulated by phospholipids. Data points represent the mean ± SEM of at least three experiments. POPC, 1-palmitoyl-2-oleoyl-sn-glycero-3-phosphocholine. POPS, 1-palmitoyl-2-oleoyl-sn-glycero-3-phospho-L-serine.

Fig. S3 Cryo-EM single particle analysis of ctDnf1p-Cdc50p with AMPPCP

a, Representative image after motion correction. **b,** Representative results of 2D classification. **c,** Workflow of the single particle analysis. **d,** Local resolution map of the final sharpened map, shown with (left) and without (right) the nanodisc. **e,** Fourier shell correlation (FSC) curve with estimated resolution according to the gold standard.

Fig. S4 Cryo-EM single particle analysis of ctDnf1p-Cdc50p with BeF_3^-.

a, Representative image after motion correction. **b,** Representative results of 2D classification. **c,** Workflow of the single particle analysis. **d,** Local resolution map of the final sharpened map shown with (left) and without (right) the nanodisc. **e,** Fourier shell correlation (FSC) curve with estimated resolution according to the gold standard.

Fig. S5 Examples of the fit of models into the density map.

a-g, Density map and model in selected regions of each domain and TMs. Residues at the beginning and end of each polypeptide segment are indicated. **h,** Density map and model of BeF_3^-, Mg^{2+}, and D606 in the E1-ATP structure. **i,** Density map and model of AMPPCP, Mg^{2+}, and D606 in the E1-ATP structure.

Fig. S6 Comparison of ctDnf1p-Cdc50p structures in the E1-ATP and E2P states.

a, Overlay of the E1-ATP and E2P structures by superimposing TMs 3-10 of ctDnf1p and ctCdc50p (grey). The A domain is yellow in E1-ATP and blue in E2P. The N domain is red in E1-ATP and cyan in E2P. TM2 is purple in E1-ATP and TMs 1 and 2 are green in E2P. **b,** same as **a**, except the N domains are omitted for clarity. The motion distance of the A domain between the E1-ATP an E2P states is labeled. **c,** same as **a**, except the A domains are omitted. The movement of the N domain between the E1 and E2 states is indicated. **d,** same as **a**, except rotating by 90 degrees and the A and N domains are omitted to show the movements of TMs 1 and 2 between the E1-ATP and E2P states.

Fig. S7 Comparison of AMPPCP in different E1-ATP structures

a-d, AMPPCP conformations from different P-type ATPases are shown as sticks. The protein structures from which AMPPCP are extracted are labeled.

Fig. S8 Comparison of phospholipid binding at E2-site1 in ctDnf1p and hATP8A1

a, Lipid binding at E2-site1 of ctDnf1p. The protein is shown as tan ribbon representation. The density of the lipid is shown as a grey mesh. The lipid (green) and its interacting residues (tan) are shown as sticks. **b,** Lipid binding in the E2Pi-PL structure of hATP8A1 (PDB ID: 6K7M, EMDB number: 9941). The protein is colored purple. The lipid is yellow. **c,** Superimposition of the two lipid binding sites. The yellow arrow and green arrow indicate the extension directions of the lipid acyl chains in hATP8A1 and ctDnf1p, respectively.

Fig. S9 Comparison of E1-site1 among P4-ATPase structures

a, E1-site1 in ctDnf1p. The density of the possible phospholipid substrate is shown as a grey mesh. The conserved serine residue is labeled. **b-c,** same as in **a**, except showing scDrs2p and hATP8A1, respectively.

Fig. S10 Interaction of the two terminal segments of ctCdc50p with ctDnf1p on the cytoplasmic side.

The N-terminal and C-terminal segments of ctCdc50p are colored yellow. The rest of ctCdc50p is pink. The ctDnf1p fragments that interact with ctCdc50p are colored red. The rest of ctDnf1p is tan. Interacting segments and TMs are labeled.

Fig. S11 A common lipid binding site in E1-ATP and E2P

a-b, Lipid binding site in E1-ATP (**a**) and E2P (**b**). The density of the lipid is shown as grey meshes. The lipid molecules are shown as sticks. **c,** Electrostatic potential surfaces of E1-ATP (left) and E2P (right), showing the lipid binding environment. The lipid molecules are shown as sticks. The surfaces showing here are on the opposite side of the surfaces showing in Fig. 2**b** and **d**.

Fig. S12 Model of phospholipid flipping by P4-ATPases

a, Cartoon drawing of E1-ATP. Domains are labeled and colored as in Fig 1**a**. The membrane is colored grey. The lipid molecules with light green heads are arranged to show the distortion of bilayers. The lipid molecule with the dark green head represents a substrate that is entering the transport pathway via E1-site2. **b,** Cartoon drawing of E2P. The lipid substrate is trapped in E2-site1. **c,** As in **a**, with the lipid bound at E1-site1. **d,** As in **b**, but the lipid substrate has been flipped to the cytosolic leaflet and waits at E2-site2 to be released.

Fig. S13 The "hydrophobic gate" residue I554 in cfDnf1p

a, Top view of the I554 and its interacting residues in E2P. The interacting residues are shown as sticks. **b,** Top view of I554 in E1-ATP. TM4 is in the same orientation as it is in **a**.

Table S1 Cryo-EM data collection, refinement and validation statistics

	ctDnf1p-Cdc50p with AMPPCP (EMDB-0873) (PDB 6LCR)	ctDnf1p-Cdc50p with BeF_3^- (EMDB-0872) (PDB 6LCP)
**Data collection and processing**		
Magnification	130,000	130,000
Voltage (kV)	300	300
Electron exposure (e–/Å^2)	57.5	57.5
Defocus range (μm)	-1.0 to -2.0	-1.0 to 2.0
Pixel size (Å)	1.055	1.055
Symmetry imposed	C1	C1
Initial particle images (no.)	2,397,258	2,820,251
Final particle images (no.)	272,912	249,694
Map resolution (Å)	3.40	3.48
FSC threshold 0.143		
Map resolution range (Å)	3.3-8.2	3.3-7.8
**Refinement**		
Model resolution (Å)	3.4	3.5
Map sharpening B factor (Å^2)	-94	-84
<u>Model composition</u>		
Non-hydrogen atoms	11590	12406
Protein residues	1420	1514
Ligands	17	18
<u>B factors (Å^2)</u>		
Protein	41.2	50.0
Ligand	45.6	51.5
<u>R.m.s. deviations</u>		
Bond lengths (Å)	0.012	0.010
Bond angles (°)	1.055	1.088
<u>Validation</u>		
MolProbity score	2.16	2.35
Clashscore	15.5	18.1
Poor rotamers (%)	0.31	0.25
<u>Ramachandran plot</u>		
Favored (%)	89.8%	88.1%
Allowed (%)	10.2%	11.7%
Disallowed (%)	0%	0.2%

Fig S1

```
CtDNF1      MAPPQEEG-GG-----------------------------------------------------------------------------------   10
CtDRS2      MSGRPPPG-AASSSHHN--------------P-NE-DLLLDLDN-DQPIYNSGQRSALTDDDLMRSHNYDQDGIA-------------------   57
ScDRS2      MNDDRETP-PK----RK--------------P-GEDDTLFDIDFLDDTTSHSGSRSKVTNS-------HANANYIPPSHVLPEETIDLDADDDN   67
ScDNF1      MSGTFHGD-GH----------------APMSP-FE-DTFQFEDN-SS-----N----------------EDTHIA-------------------   34
ScDNF2      MSSPSKPT-SPFVDDIEHESGSASNGLSSMSP-FD-DSFQFEKP-SS-----A----------------HGNIEVA------------------   51
BtATP8A2    MSRAT-----------------------------------------------------------------------------------------    5
HsATP8A1    MPTMRRTV-----------------------------------------------------------S--------------------------    9
HsATP8A2    MLNGAGLD----------------------------------------------------KALKMSLPRRSRIRSS------------------   24
HsATP11A    MDCSLVRTLVHR----------------------------------------------------------------------------------   12
HsATP11C    MQMVPSL-PPAS----------------------------------------------------------------------------------   11
HsATP8B1    MSTERDSE-TTFDEDSQ--------------P-ND-EVVPYSDD--------ET----------------------------------------   29
HsATP8B2    MTVPKEMP-EKWARAQA--------------PPS------------------WS----------------------------------------   21
HsATP10A    MEREPAGT-EEP----------------------------------------------------------------------------------   11
AtALA2      MK--------------------------------------------------------------------------------------------    2
AtALA10     MAGPSRRR-RRLH---------------------------------------------------LS----------------------------   14


CtDNF1      ----------------------------------------------------------------------------------------------    -
CtDRS2      --------------------------------PSRPSVSYD-DFIGSSSSQPHRSAGRPPPS------SGLSAPGPSSRPVGPY--STAEINRQ  110
ScDRS2      IENDVHENLFMSNNHDDQTSWNANRFDSDAYQP-------------------QSLRAVKPPGLFARFGNGLKNAFTFKRKKGP---ESFEMNHY  139
ScDNF1      -----------------------------------P-THFD----------------------------DGATSNKYSRPQVSFNDETPKNKR-   63
ScDNF2      -----------------------------------K-T-------------------------------GGSVLKRQSKPMKDI--STPDLSKV   76
BtATP8A2    ----------------------------------------------------------------------------------------------    -
HsATP8A1    -----------------------------------E---IRSRAEGYE----------------------------------------------   19
HsATP8A2    ---------------------------------VGP---VRSS-LGYK----------------------------------------------   35
HsATP11A    ------------------------------------------Y---------------------------------------------------   13
HsATP11C    ------------------------------------------E---------------------------------------------------   12
HsATP8B1    ----------------------------------------------------------------------------------------------    -
HsATP8B2    ----------------------------------------------------------------------------------------------    -
HsATP10A    ------------------------------------------GPPGRR----------------------------------------------   17
AtALA2      ----------------------------------------------------------------------------------------------    -
AtALA10     -----------------------------------K---IYSYTCGKS----------------------------------------------   24


CtDNF1      ----------------------------------------------------------------------------NG-TE--LSMQRSRWATR   25
CtDRS2      -YSQT-SDLGNYQRYADDYDDYPDEGTSYYQHGGAGGEPSSGRANA----RQRNSVLSLGG-GFL-GRVKNRLGMGQGYSEMDLPLTESRTGHR  196
ScDRS2      -NAVT-NNELDDNYLDS----------------------RNKFNI---------------------------------------KILFNRYILR  170
ScDNF1      --EDA-EEFT-----------------------------FNDDTEYDNHSFQPTPKLNNGSGTFDDVE-LDNDSGEPH-TNY-DGMKRFRMGTK  122
ScDNF2      TFDGI-DDYSNDNDIND-DDELNGKKTEIHE--------HENEVDDDLHSFQATPMPNTG--GFEDVE-LDNNEGSNNDSQADHKLKRVRFGTR  157
BtATP8A2    -------------S--------------------------------------------------------------------------------    6
HsATP8A1    ---KT-DDVSEKTS--------------------------------------------------------------------------------   29
HsATP8A2    ---KAEDEMSRATS--------------------------------------------------------------------------------   46
HsATP11A    -------------C--------------------------------------------------------------------------------   14
HsATP11C    -------------C--------------------------------------------------------------------------------   13
HsATP8B1    ----E-----DELD---------DQGSAVEP--------EQNRVNR--------------------------------------EAEENREPFR   59
HsATP8B2    ----R-----KKPS--------------------------------------------------------------------------------   26
HsATP10A    ---RR-R--EG-----------------------------------------------------------------------------------   22
AtALA2      ----------------------------------------------------------------------------------------------    -
AtALA10     ---SF-Q--EDHSN--------------------------------------------------------------------------------   32

                                                                                             A domain
CtDNF1      RLTVKSGARKRLSLMTR--------------------AQAKNSATEKRQSGV--TDDGSPAADGD-QKEGSISSSNNGGSAPRKLYFNLPLPPE   96
CtDRS2      TDT------------------GSGEIPAQPEKKKFDFGNFRFGFGRS---------------KPD-P----------STLGPRIIYLNNPP---  243
ScDRS2      KN------------------------------------------------------------VGDAE----------GNGEPRVIHINDSL---  191
ScDNF1      RNKKGN------PIMGRSKTLK------------WARKNIPNPFED----FT--KDDIDPGAI-------------NRAQELRTVYYNMPLPKD  179
ScDNF2      RNKSGR------IDINRSKTLK------------WAKKNFHNAIDE----FSTKEDSLENSAL------------QNRSDELRTVYYNLPLPED  217
BtATP8A2    --------------------------------------------------------------VGD-Q----------LDVPARTIYLNQPH---   24
HsATP8A1    --------------------------------------------------------------LAD-Q------------EEVRTIFINQPQ---   45
HsATP8A2    --------------------------------------------------------------VGD-Q----------LEAPARTIYLNQPH---   64
HsATP11A    --------------------------------------------------------------AGE-E----------NWVDSRTIYVGHREPP-   34
HsATP11C    --------------------------------------------------------------AGE-E----------KRVGTRTVFVGNH-PV-   32
HsATP8B1    KE------------------------------------------------------------CTW-Q----------VKANDR-KYHEQPHFM-   80
HsATP8B2    --------------------------------------------------------------WG--------------TEEERRARANDRE---   41
HsATP10A    --------------------------------------------------------------------------------RTRTVRSNLLPPPG   36
AtALA2      ----------------------------------------------------------------------------------RFVYINDDE---   11
AtALA10     --------------------------------------------------------------IGG-------------PGFSRVVYCNEPGS--   49

                    A domain                                 TM1                                 TM2
CtDNF1      LKDE-----------EGHPIQQFPRNKIRTAKYTPLSFIPKNLWFQFHNIANIFFLFLVILVIFPIFGGVNPGLNSVPLIVIITVTAIKDAIED  179
CtDRS2      ----------------ANAANKYVDNHVSTAKYNFATFLPKFLFEQFSKFANIFFLFTAGLQQIPGLSPTNRYTTIGPLIVVLLVSAGKELVED  321
ScDRS2      ----------------ANSSFGYSDNHISTTKYNFATFLPKFLFQEFSKYANLFFLCTSAIQQVPHVSPTNRYTTIGTLLVVLIVSAMKECIED  269
ScDNF1      MIDE-----------EGNPIMQYPRNKIRTTKYTPLTFLPKNILFQFHNFANVYFLVLIILGAFQIFGVTNPGLSAVPLVVIVIITAIKDAIED  262
ScDNF2      MLDE-----------DGLPLAVYPRNKIRTTKYTPLTFFPKNILFQFHNFANIYFLILLILGAFQIFGVTNPGFASVPLIVIVIITAIKDGIED  300
BtATP8A2    -------------------LNKFCDNQISTAKYSVVTFLPRFLYEQIRRAANAFFLFIALLQQIPDVSPTGRYTTLVPLIIILTIAGIKEIVED   99
HsATP8A1    -------------------LTKFCNNHVSTAKYNIITFLPRFLYSQFRRAANSFFLFIALLQQIPDVSPTGRYTTLVPLLFILAVAAIKEIIED  120
HsATP8A2    -------------------LNKFRDNQISTAKYSVLTFLPRFLYEQIRRAANAFFLFIALLQQIPDVSPTGRYTTLVPLIIILTIAGIKEIVED  139
HsATP11A    -PGA-----------EAYIPQRYPDNRIVSSKYTFWNFIPKNLFEQFRRVANFYFLIIFLVQLI-IDTPTSPVTSGLPLFFVITVTAIKQGYED  115
HsATP11C    -SET-----------EAYIAQRFCDNRIVSSKYTLWNFLPKNLFEQFRRIANFYFLIIFLVQVT-VDTPTSPVTSGLPLFFVITVTAIKQGYED  113
HsATP8B1    -NTKFL----------CIKESKYANNAIKTYKYNAFTFIPMNLFEQFKRAANLYFLALLILQAVPQISTLAWYTTLVPLLVVLGVTAIKDLVDD  163
HsATP8B2    ----------------YNEKFQYASNCIKTSKYNILTFLPVNLFEQFQEVANTYFLFLLILQLIPQISSLSWFTTIVPLVLVLTITAVKDATDD  119
HsATP10A    AEDPAAGAAKGERRRRRGCAQHLADNRLKTTKYTLLSFLPKNLFEQFHRPANVYFVFIALLNFVPAVNAFQPGLALAPVLFILAITAFRDLWED  130
AtALA2      -----------------ASKELCCDNRISNRKYTLWNFLPKNLWEQFSRFMNQYFLLIACLQLWSLITPVNPASTWGPLIFIFAVSASKEAWDD   88
AtALA10     ---------------PAAERRNYAGNYVRSTKYTVASFFPKSLFEQFRRVANFYFLVTGILSL-TDLSPYGAVSALLPLALVISATMVKEGIED  127
```

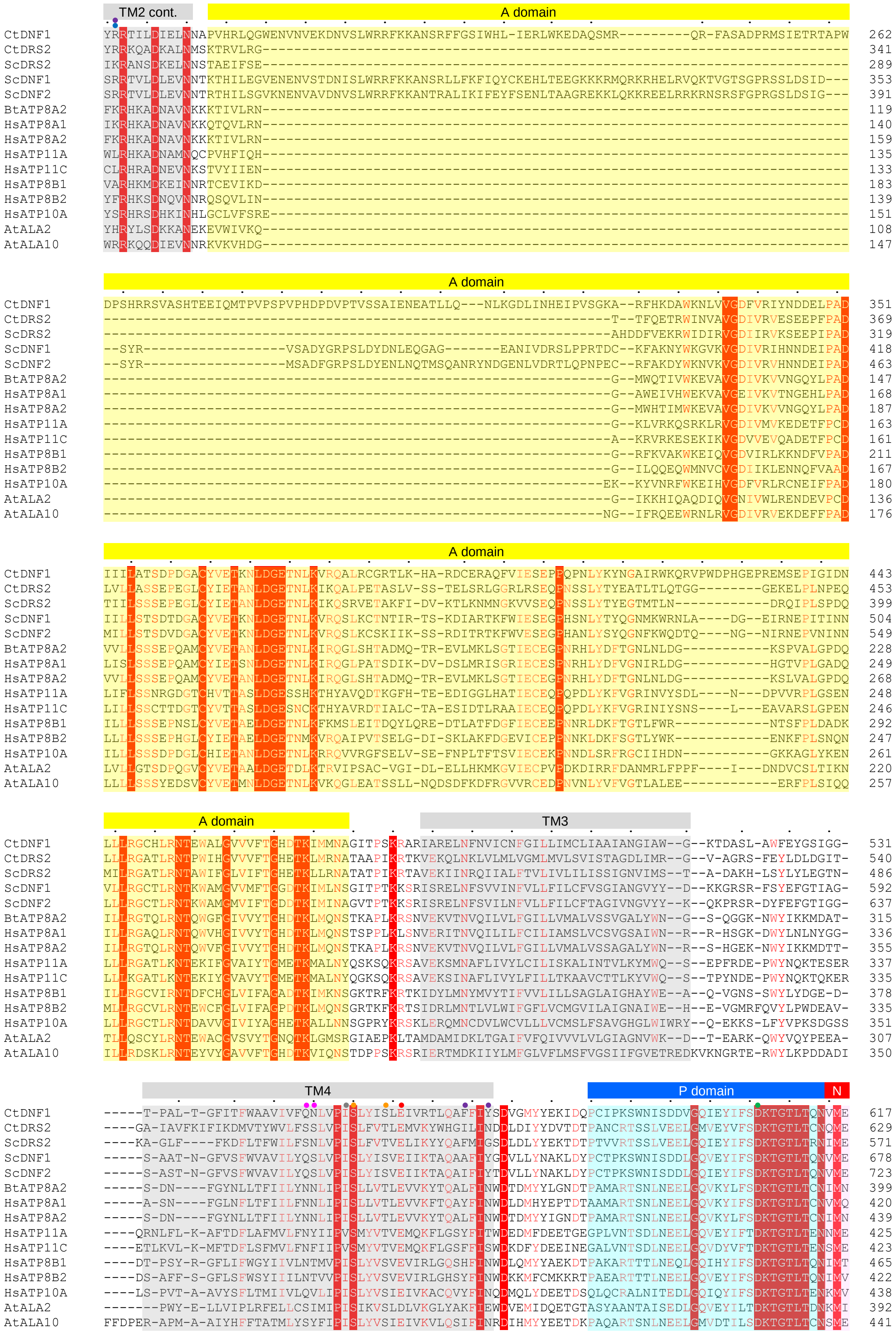
TM2 cont.
A domain
TM3
TM4
P domain
N

N domain

```
CtDNF1    FKKATINGQPYGEAYTEAQAG--M--DRRRGINVEEEA---------------KVIREEIAAAKVR-----------------------AIRGL  669
CtDRS2    FKACSIAGVMYAESVPEDR-V--A--TIEDGV---------------------------------------------------------------  656
ScDRS2    FKSCSIAGHCYIDKIPEDK-T--A--TVEDGI---------------------------------------------------------------  598
ScDNF1    FKKCTINGVSYGRAYTEALAG--L--RKRQGIDVETEG---------------RREKAEIAKDRDT-----------------------MIDEL  730
ScDNF2    FKKCTINGVSYGRAYTEALAG--L--RKRQGVDVESEG---------------RREKEEIAKDRET-----------------------MIDEL  775
BtATP8A2  FKKCSIAGVTYGHFPELTR-E--P--SSDDFS--R------------------------------------------------------------  427
HsATP8A1  FKKCTIAGVAYGHVPEPEDYG--C--SPDEWQ---------------------------------------------------------------  448
HsATP8A2  FKKCSIAGVTYGHFPELAR-E--P--SSDDFC--R------------------------------------------------------------  467
HsATP11A  FKECCIEGHVYVPHVICNGQV--L--PESSGID--------------------------------------------------------------  454
HsATP11C  FIECCIDGHKYKGVTQ-EVDG--L--SQTDGTL--------------------------------------------------------------  451
HsATP8B1  FKKCCINGQIYGDHRDASQ-H--NH-NKIEQVDFS------------------------------------------------------------  496
HsATP8B2  FNKCSINGHSYGDVFDVLG-HKAELGERPEPVDFS------------------------------------------------------------  456
HsATP10A  FRRCTVSGVEYSHDANAQR-L-----ARYQEADSEEEEVVPRGGSVSQRGSIGS------HQSVRVVHRTQSTKSHRRTGSRAEAKRASMLSKH  520
AtALA2    FRRCCIGGIFYGNENGDA-----------------------------------------------------------------------------  410
AtALA10   FIKCSIAGKAYGRGITEVERA--M--AVRSGGS------------------PLV------NEDLDV-----------------------VVD--  482
```

N domain

```
CtDNF1    RELHDNPYLHDEDMTFIA-PDFVEDLAGK---------------NGPEQQQATEHFMLALALCHTVVAEKQ----------------------  724
CtDRS2    ------------EVGIHDFKRLKDNLKN-----------------GHPTAQAIDHFLTLLATCHTVIPEQK----------------------  698
ScDRS2    ------------EVGYRKFDDLKKLND-----------------PSDEDSPIINDFLTLLATCHTVIPEFQ----------------------  641
ScDNF1    RALSGNSQFYPEEVTFVS-KEFVRDLKGA---------------SGEVQQRCCEHFMLALALCHSVLVEAN----------------------  785
ScDNF2    RSMSDNTQFCPEDLTFVS-KEIVEDLKGS---------------SGDHQQKCCEHFLLALALCHSVLVEPN----------------------  830
BtATP8A2  -----IPPPPSDSCDFDD-PRLLKNIED-----------------HHPTAPCIQEFLTLLAVCHTVVPERD----------------------  475
HsATP8A1  -----NS-QFGDEKTFSD-SSLLENLQN-----------------NHPTAPIICEFLTMMAVCHTAVPERE----------------------  495
HsATP8A2  -----MPPPCSDSCDFDD-PRLLKNIED-----------------RHPTAPCIQEFLTLLAVCHTVVPEKD----------------------  515
HsATP11A  ---------------MID------SSPS------------------V-NGREREELFFRALCLCHTVQVKDDDSVDGP----------------  492
HsATP11C  --------------TYFD------K---------------------VDKNREELFLRALCLCHTVEIKTNDAVD--------------------  484
HsATP8B1  -----WNTYADGKLAFYD-HYLIEQIQS-----------------G--KEPEVRQFFFLLAVCHTVMVDR-----------------------  541
HsATP8B2  -----FNPLADKKFLFWD-PSLLEAVKI-----------------G---DPHTHEFFRLLSLCHTVMSEEK----------------------  501
HsATP10A  TA---FSSPM-EKDITPD-PKLLEKVSECDKSLAVARHQEHLLAHLSPELSDVFDFFIALTICNTVVVTSPDQ---PRTKVRVRFELKSPVKTI  606
AtALA2    ---------------LKD-AQLLNAITS--------------------GSTDVIRFLTVMAICNTVLPVQ----------------------  444
AtALA10   -----QSGPKVKGFNFED-ERVMNGNWV-----------------RQPEAAVLQKFFRLLAVCHTAIPETD----------------------  530
```

N domain

```
CtDNF1    ---------------------------------------------------------------------------PGDPPKMIFKAQSP  738
CtDRS2    -----------------------------------------------------------------------------DSGEIKYQASSP  710
ScDRS2    -----------------------------------------------------------------------------SDGSIKYQAASP  653
ScDNF1    ---------------------------------------------------------------------------PDNPKKLDLKAQSP  799
ScDNF2    ---------------------------------------------------------------------------KDDPKKLDIKAQSP  844
BtATP8A2  ------------------------------------------------------------------------------GDSIVYQASSP  486
HsATP8A1  ------------------------------------------------------------------------------GDKIIYQAASP  506
HsATP8A2  ------------------------------------------------------------------------------GDNIIYQASSP  526
HsATP11A  ------------------------------------------------------------------------RKSPDGGKSCVYISSSP  509
HsATP11C  ------------------------------------------------------------------------G--ATESAELTYISSSP  499
HsATP8B1  -----------------------------------------------------------------------------TDGQLNYQAASP  553
HsATP8B2  -----------------------------------------------------------------------------NEGELYYKAQSP  513
HsATP10A  EDFLRRFTPSCLTSGCSSIGSLAANKSSHKLGSSFPSTPSSDGMLLRLEERLGQPTSAIASNGYSSQADNWASELAQE--QESERELRYEAESP  698
AtALA2    -----------------------------------------------------------------------------SKAGDIVYKAQSQ  457
AtALA10   -----------------------------------------------------------------------------EESGNVSYEAESP  543
```

N domain

```
CtDNF1    DEAALVATARDMGFTVLGMSDGGIN--VNV------MGKDMHFPVLSIIEFNSSRKRMSTIVRM-P------DGRILLFCKGADSVIYSRLKKG  817
CtDRS2    DEGALVEGAVQLGYRFLARKPRAVI--ITV------NGQQLEYELLAVCEFNSTRKRMSTIYRC-P------DGKIRIYCKGADTVILERLNDQ  789
ScDRS2    DEGALVQGGADLGYKFIIRKPNSVTVLLEE------TGEEKEYQLLNICEFNSTRKRMSAIFRF-P------DGSIKLFCKGADTVILERLDDE  734
ScDNF1    DEAALVATARDVGFSFVGKTKKGLI--IEM------QGIQKEFEILNILEFNSSRKRMSCIVKI-PGLNPGDEPRALLICKGADSIIYSRLSRQ  884
ScDNF2    DESALVSTARQLGYSFVGSSKSGLI--VEI------QGVQKEFQVLNVLEFNSSRKRMSCIIKI-PGSTPKDEPKALLICKGADSVIYSRLDRT  929
BtATP8A2  DEAALVKGARKLGFVFTARTPYSVI--IEA------MGQEQTFGILNVLEFSSDRKRMSVIVRT-P------SGQLRLYCKGADNVIFERLSKD  565
HsATP8A1  DEGALVRAAKQLNFVFTGRTPDSVI--IDS------LGQEERYELLNVLEFTSARKRMSVIVRT-P------SGKLRLYCKGADTVIYDRLAET  585
HsATP8A2  DEAALVKGAKKLGFVFTARTPFSVI--IEA------MGQEQTFGILNVLEFSSDRKRMSVIVRT-P------SGRLRLYCKGADNVIFERLSKD  605
HsATP11A  DEVALVEGVQRLGFTYLRLKDNYME--ILN------RENHIERFELLEILSFDSVRRRMSVIVKS-A------TGEIYLFCKGADSSIFPRVIEG  589
HsATP11C  DEIALVKGAKRYGFTFLGNRNGYMR--VEN-----QRKEIEEYELLHTLNFDAVRRRMSVIVKT-Q------EGDILLFCKGADSAVFPRVQNH  579
HsATP8B1  DEGALVNAARNFGFAFLARTQNTIT--ISE------LGTERTYNVLAILDFNSDRKRMSIIVRT-P------EGNIKLYCKGADTVIYERLHRM  632
HsATP8B2  DEGALVTAARNFGFVFRSRTPKTIT--VHE------MGTAITYQLLAILDFNNIRKRMSVIVRN-P------EGKIRLYCKGADTILLDRLHHS  592
HsATP10A  DEAALVYAARAYNCVLVERLHDQVS--VEL----PHLG-RLTFELLHTLGFDSVRKRMSVVIRH-PL-----TDEINVYTKGADSVVMDLLQPC  779
AtALA2    DEDALVIAASKLHMVFVGKNANLLE--IRF------NGSVIRYEVLEILEFTSDRKRMSVVVKDCQ------NGKIILLSKGADEAILPYARA-  536
AtALA10   DEAAFVVAAREFGFEFFNRTQNGIS--FRELDLVSGEKVERVYRLLNVLEFNSTRKRMSVIVRD-D------DGKLLLLSKGADNVMFERLAKN  628
```

N domain | P

```
CtDNF1    E--------QADMRRETAQHLEMFAVEGLRTLCIAERELSEEEYREWRREHDLAATAL-ENREEKLEEVADKIERDLTLLGGTAIEDRLQDGVP  902
CtDRS2    N----------PHVDQTLRHLEEYASEGLRTLCLAFREVPEQEFQEWYQVYDKAQTTVGGTRAQELDKAAEIIEKDFYLLGATAIEDRLQDGVP  873
ScDRS2    A----------NQYVEATMRHLEDYASEGLRTLCLAMRDISEGEYEEWNSIYNEAATTL-DNRAEKLDEAANLIEKNLILIGATAIEDKLQDGVP  818
ScDNF1    SG-----SNSEAILEKTALHLEQYATEGLRTLCIAQRELSWSEYEKWNEKYDIAAASL-ANREDELEVVADSIERELILLGGTAIEDRLQDGVP  972
ScDNF2    QN-------DATLLEKTALHLEEYATEGLRTLCLAQRELTWSEYERWVKTYDVAAASV-TNREEELDKVTDVIERELILLGGTAIEDRLQDGVP  1015
BtATP8A2  S----------KYMEETLCHLEYFATEGLRTLCVAYADLSERDYEEWLKVYQEASTIL-KDRAQRLEECYEIIEKNLLLLGATAIEDRLQAGVP  648
HsATP8A1  S----------KYKEITLKHLEQFATEGLRTLCFAVAEISESDFQEWRAVYQRASTSV-QNRLLKLEESYELIEKNLQLLGATAIEDKLQDQVP  668
HsATP8A2  S----------KYMEETLCHLEYFATEGLRTLCVAYADLSENEYEEWLKVYQEASTIL-KDRAQRLEECYEIIEKNLLLLGATAIEDRLQAGVP  688
HsATP11A  ------------KVDQIRARVERNAVEGLRTLCVAYKRLIQEEYEGICKLLQAAKVAL-QDREKKLAEAYEQIEKDLTLLGATAVEDRLQEKAA  670
HsATP11C  ------------EIELTKVHVERNAMDGYRTLCVAFKEIAPDDYERINRQLIEAKMAL-QDREEKMEKVFDDIETNMNLIGATAVEDKLQDQAA  660
HsATP8B1  N----------PTKQETQDALDIFANETLRTLCLCYKEIEEKEFTEWNKKFMAASVAS-TNRDEALDKVYEEIEKDLILLGATAIEDKLQDGVP  715
HsATP8B2  T-----------QELLNTTMDHLNEYAGEGLRTLVLAYKDLDEEYYEEWAERRLQASLAQ-DSREDRLASIYEEVENNMMLLGATAIEDKLQQGVP  676
HsATP10A  SSVDARGRHQKKIRSKTQNYLNVYAAEGLRTLCIAKRVLSKEEYACWLQSHLEAESSL-ENSEELLFQSAIRLETNLHLLGATGIEDRLQDGVP  872
AtALA2    ---------GQQ-TRTIGDAVEHYSQLGLRTLCLAWRELEENEYLEWSVKFKEASSLL-VDREWRIAEVCQRLEHDLYILGVTAIEDRLQDGVP  619
AtALA10   ---------GRQFEAKTQEHVNQYADAGLRTLVLAYREVDENEYIEFNKSFNEAKASVSEDREALIDEITDKMERDLILLGATAVEDKLQNGVP  713
```

```
                                                    P domain
CtDNF1     DTIALLADAGIKLWVLTGDKVETAINIGFSCNLLNNDMD--LLRL-QVNESDAST-E--DDYLQLA---------------E------EQLKTN   969
CtDRS2     ETIHTLQEAGIKVWVLTGDRQETAINIGMSCKLLSEDMM--LLII-NEETAEATR----DNIQ-----------------------------KK   931
ScDRS2     ETIHTLQEAGIKIWVLTGDRQETAINIGMSCRLLSEDMN--LLII-NEETRDDTE----RNLL-----------------------------EK   876
ScDNF1     DCIELLAEAGIKLWVLTGDKVETAINIGFSCNLLNNEME--LLVI-KTTGDDVKEFG--SEPSEIV---------------D------ALLSKY  1040
ScDNF2     DSIALLAEAGIKLWVLTGDKVETAINIGFSCNVLNNDME--LLVV-KASGEDVEEFG--SDPIQVV---------------N------NLVTKY  1083
BtATP8A2   ETIATLLKAEIKIWVLTGDKQETAINIGYSCRLVSQNMA--LILL-KEDSLDATR----AAIT-----------------------------QH   706
HsATP8A1   ETIETLMKADIKIWILTGDKQETAINIGHSCKLLKKNMG--MIVI-NEGSLDGTR----ETLS-----------------------------RH   726
HsATP8A2   ETIATLLKAEIKIWVLTGDKQETAINIGYSCRLVSQNMA--LILL-KEDSLDATR----AAIT-----------------------------QH   746
HsATP11A   DTIEALQKAGIKVWVLTGDKMETAAATCYACKLFRRNTQ--LLEL-TTKRIEEQS------LH------------------DVLFELSKTVLRH   737
HsATP11C   ETIEALHAAGLKVWVLTGDKMETAKSTCYACRLFQTNTE--LLEL-TTKTIEESE----RKEDRLH---------------ELLIEYRKKLLHE   732
HsATP8B1   ETISKLAKADIKIWVLTGDKKETAENIGFACELLTEDTT--IC-YGE-DI-N-----------SL----------------L------HARMEN   771
HsATP8B2   ETIALLTLANIKIWVLTGDKQETAVNIGYSCKMLTDDMT--EVFIVTGHTVLEVR----EELRKA----------------R------EKMMDS   742
HsATP10A   ETISKLRQAGLQIWVLTGDKQETAVNIAYACKLLDHDEE--VITL-NATSQEACA----ALLDQCLCYVQSRGLQRAPEKTK------GKVSMR   953
AtALA2     ETIETLRKAGINFWMLTGDKQNTAIQIALSCNFISPEPKGQLLMI-DGKTEEDVS----RSLERVL----------------------------   680
AtALA10    ECIDKLAQAGIKIWVLTGDKMETAINIGFASSLLRQEMK--QIII-NLETPQIKSLEKSGGKDEIE------------LASR------ESVVMQ   786

                                                    P domain
CtDNF1     L-ERFNMTGDDEELK--RARKDHNAPSPTYALVIDGFTLRWVL-----------------------------------S-------DSLKQKFL  1018
CtDRS2     LDAIR-------------AQEHGTVEMGTLALVIDGKSLTYAL-----------------------------------E-------RDLEKMFL   970
ScDRS2     INALN-------------EHQLSTHDMNTLALVIDGKSLGFAL-----------------------------------E-------PELEDYLL   915
ScDNF1     LKEYFNLTGSEEEIF--EAKKDHEFPKGNYAIVIDGDALKLAL-----------------------------------YG------EDIRRKFL  1091
ScDNF2     LREKFGMSGSEEELK--EAKREHGLPQGNFAVIIDGDALKVAL-----------------------------------NG------EEMRRKFL  1134
BtATP8A2   CADL----------------GSLLGKENDAALIIDGHTLKYAL-----------------------------------S-------FEVRRSFL   742
HsATP8A1   CTTL----------------GDALRKENDFALIIDGKTLKYAL-----------------------------------T-------FGVRQYFL   762
HsATP8A2   CTDL----------------GNLLGKENDVALIIDGHTLKYAL-----------------------------------S-------FEVRRSFL   782
HsATP11A   SGSLT-----R------DNLSGLSADMQDYGLIIDGAALSLIM-----------------------------------KPREDGSSGNYRELFL   785
HsATP11C   FP-KS-----T------RSFKKAWTEHQEYGLIIDGSTLSLIL-----------------------------------NSSQDSSSNNYKSIFL   779
HsATP8B1   QRNRGGVY---AKFAPPV-QESFFPPGGNRALIITGSWLNEILLEKKTKRNKILKLFPRTEEERRMRTQSKRRLEAKK-------EQRQKNFV   854
HsATP8B2   SRSVGNGFTYQDKLSSSKLTSVLEAVAGEYALVINGHSLAHAL-----------------------------------E-------ADMELEFL   794
HsATP10A   FSSLC------------P-PSTSTASGRRPSLVIDGRSLAYAL-----------------------------------E-------KNLEDKFL   992
AtALA2     -----------------LTMRITASEPKDVAFVIDGWALEIAL-----------------------------------K-------H-HRKDFV   714
AtALA10    LQEGK------------ALLAASGASSEAFALIIDGKSLTYAL-----------------------------------E-------DEIKKMFL   826

                                         P domain                                        TM5
CtDNF1     LLCKQCKSVLCCRVSPAQKAAVVSMVKNGL-DVMTLSIGDGANDVAMIQEADVGVGIAGEEGRQAVMSSDFAIGQFRFLQRLVLVHGRWSYRRL  1111
CtDRS2     DLAIMCKAVICCRVSPLQKAMVVKLVKKYQKESILLAIGDGANDVSMIQAAHIGIGISGVEGLQAARSADVSIAQFRYLRKLLLVHGAWSYHRV  1064
ScDRS2     TVAKLCKAVICCRVSPLQKALVVKMVKRKS-SSLLLAIGDGANDVSMIQAAHVGVGISGMEGMQAARSADIAVGQFKFLKKLLLVHGSWSYQRI  1008
ScDNF1     LLCKNCRAVLCCRVSPSQKAAVVKLVKDSL-DVMTLAIGDGSNDVAMIQSADVGIGIAGEEGRQAVMCSDYAIGQFRYLARLVLVHGRWSYKRL  1184
ScDNF2     LLCKNCKAVLCCRVSPAQKAAVVKLVKKTL-DVMTLAIGDGSNDVAMIQSADVGVGIAGEEGRQAVMCSDYAIGQFRYVTRLVLVHGKWCYKRL  1227
BtATP8A2   DLALSCKAVICCRVSPLQKSEIVDVVKKRV-KAITLAIGDGANDVGMIQTAHVGVGISGNEGMQATNNSDYAIAQFSYLEKLLLVHGAWSYNRV   835
HsATP8A1   DLALSCKAVICCRVSPLQKSEVVEMVKKQV-KVVTLAIGDGANDVSMIQTAHVGVGISGNEGLQAANSSDYSIAQFKYLKNLLMIHGAWNYNRV   855
HsATP8A2   DLALSCKAVICCRVSPLQKSEIVDVVKKRV-KAITLAIGDGANDVGMIQTAHVGVGISGNEGMQATNNSDYAIAQFSYLEKLLLVHGAWSYNRV   875
HsATP11A   EICRSCSAVLCCRMAPLQKAQIVKLIKFSKEHPITLAIGDGANDVSMILEAHVGIGVIGKEGRQAARNSDYAIPKFKHLKKMLLVHGHFYYIRI   879
HsATP11C   QICMKCTAVLCCRMAPLQKAQIVRMVKNLKGSPITLSIGDGANDVSMILESHVGIGIKGKEGRQAARNSDYSVPKFKHLKKLLLAHGHLYYVRI   873
HsATP8B1   DLACECSAVICCRVTPKQKAMVVDLVKRYK-KAITLAIGDGANDVNMIKTAHIGVGISGQEGMQAVMSSDYSFAQFRYLQRLLLVHGRWSYIRM   947
HsATP8B2   ETACACKAVICCRVTPLQKAQVVELVKKYK-KAVTLAIGDGANDVSMIKTAHIGVGISGQEGIQAVLASDYSFSQFKFLQRLLLVHGRWSYLRM   887
HsATP10A   FLAKQCRSVLCCRSTPLQKSMVVKLVRSKL-KAMTLAIGDGANDVSMIQVADVGVGISGQEGMQAVMASDFAVPKFRYLERLLILHGHWCYSRL  1085
AtALA2     ELAILSRTAICCRVTPSQKAQLVEILKS-C-DYRTLAIGDGGNDVRMIQQADIGVGISGREGLQAARAADYSIGRFRFLKRLILVHGRYSYNRT   806
AtALA10    DLATSCASVICCRSSPKQKALVTRLVKSGT-GKTTLAIGDGANDVGMLQEADIGVGISGVEGMQAVMSSDIAIAQFRYLERLLLVHGHWCYSRI   919

                TM5 cont.                    TM6                                        TM7
CtDNF1     AETISNFFYKNMIWTWSIFWYQCYCNFDIAYIFEYTYILMFNLFFTSVPVILMGVLDQDVSDTVSLAVPQLYRRGIERKEWTQTKFWLYMIDGV  1205
CtDRS2     SKTILFSFYKNITLYLTQFWYTFENVFSGEVIYESWTLSFYNVFFTVLPPLVLGILDQFVSARLLDRYPQLYSLGQNNTFFRIKVFAAWIASAI  1158
ScDRS2     SVAILYSFYKNTALYMTQFWYVFANAFSGQSIMESWTMSFYNLFFTVWPPFVIGVFDQFVSSRLLERYPQLYKLGQKGQFFSVYIFWGWIINGF  1102
ScDNF1     AEMIPEFFYKNMIFALALFWYGIYNDFDGSYLYEYTYMMFYNLAFTSLPVIFLGILDQDVNDTISLVVPQLYRVGILRKEWNQRKFLWYMLDGL  1278
ScDNF2     AEMIPQFFYKNVIFTLSLFWYGIYNNFDGSYLFEYTYLTFYNLAFTSVPVILLAVLDQDVSDTVSMLVPQLYRVGILRKEWNQTKFLWYMLDGV  1321
BtATP8A2   TKCILYCFYKNVVLYIIELWFAFVNGFSGQILFERWCIGLYNVIFTALPPFTLGIFERSCSQESMLRFPQLYKITQNAEGFNTKVFWGHCINAL   929
HsATP8A1   SKCILYCFYKNIVLYIIEIWFAFVNGFSGQILFERWCIGLYNVMFTAMPPLTLGIFERSCRKENMLKYPELYKTSQNALDFNTKVFWVHCLNGL   949
HsATP8A2   TKCILYCFYKNVVLYIIELWFAFVNGFSGQILFERWCIGLYNVIFTALPPFTLGIFERSCTQESMLRFPQLYKITQNGEGFNTKVFWGHCINAL   969
HsATP11A   SELVQYFFYKNVCFIFPQFLYQFFCGFSQQTLYDTAYLTLYNISFTSLPILLYSLMEQHVGIDVLKRDPTLYRDVAKNALLRWRVFIYWTLLGL   973
HsATP11C   AHLVQYFFYKNLCFILPQFLYQFFCGFSQQPLYDAAYLTMYNICFTSLPILAYSLLEQHINIDTLTSDPRLYMKISGNAMLQLGPFLYWTFLAA   967
HsATP8B1   CKFLRYFFYKNFAFTLVHFWYSFFNGYSAQTAYEDWFITLYNVLYTSLPVLLMGLLDQDVSDKLSLRFPGLYIVGQRDLLFNYKRFFVSLLHGV  1041
HsATP8B2   CKFLCYFFYKNFAFTMVHFWFGFFCGFSAQTVYDQYFITLYNIVYTSLPVLAMGVFDQDVPEQRSMEYPKLYEPGQLNLLFNKREFFICIAQGI   981
HsATP10A   ANMVLYFFYKNTMFVGLLFWFQFFCGFSASTMIDQWYLIFFNLLFSSLPPLVTGVLDRDVPANVLLTNPQLYKSGQNMEEYRPRTFWFNMADAA  1179
AtALA2     AFLSQYSFYKSLLICFIQIFFSFISGVSGTSLFNSVSLMAYNVFYTSVPVL-VSVIDKDLSEASVMQHPQILFYCQAGRLLNPSTFAGWFGRSL   899
AtALA10    ASMICYFFYKNITFGVTVFLYEAYTSFSGQPAYNDWFLSLFNVFFSSLPVIALGVFDQDVSARFCYKFPLLYQEGVQNILFSWKRIIGWMFNGF  1013

              TM7 cont.                         TM8                         TM9
CtDNF1     YQSVMSFFIPFIFVVLTPTAAGNGLDVSERTRLGAYIAHPAVITINGYILINTYRWDWLMLLSIVLSDVFIFF-----W-TGV-Y-TATTYSAG  1291
CtDRS2     YHSILLYIGGSLFF-LGVQ-NAEGFPAG-KWVWGTAMYGAVLLTVLGKAALVTNNWTKWHVVGIPGSMLFWLV-FVGVY-GTV-A-PKLGFSME  1245
ScDRS2     FHSAIVFIGTILIYRYGFALNMHGELAD-HWSWGVTVYTTSVIIVLGKAALVTNQWTKFTLIAIPGSLLFWLI-FFPIY-ASI-F-PHANISRE  1191
ScDNF1     YQSIICFFFPYLVYHKNMIVTSNGLGLDHRYFVGVYVTTIAVISCNTYVLLHQYRWDWFSGLFIALSCLVVFA-----W-TGI-W-SSAIASRE  1364
ScDNF2     YQSVICFFFPYLAYHKNMVVTENGLGLDHRYFVGVFVTAIAVTSCNFYVFMEQYRWDWFCGLFICLSLAVFYG-----W-TGI-W-TSSSSSNE  1407
BtATP8A2   VHSLILFWFPMKALEHDT-VLANGHATD-YLFVGNIVYTYVVVTVCLKAGLETTAWTKFSHLAVWGSMLIWLV-FFGIY-STI-W-PTIPIAPD  1017
HsATP8A1   FHSVILFWFPLKALQYGT-AFGNGKTSD-YLLLGNFVYTFVVITVCLKAGLETSYWTWFSHIAIWGSIALWVV-FFGIY-SSL-W-PAIPMAPD  1037
HsATP8A2   VHSLILFWFPMKALEHDT-VLTSGHATD-YLFVGNIVYTYVVVTVCLKAGLETTAWTKFSHLAVWGSMLTWLV-FFGIY-STI-W-PTIPIAPD  1057
HsATP11A   FDALVFFFGAYFVFENTT-VTSNGQIFG-NWTFGTLVFTVMVFTVTLKLALDTHYWTWINHFVIWGSLLFYVV-FSLLW-GGVIW-PFLNY-QR  1061
HsATP11C   FEGTVFFFGTYFLFQTAS-LEENGKVYG-NWTFGTIVFTVLVFTVTLKLALDTRFWTWINHFVIWGSLAFYVF-FSFFW-GGIIW-PFLKQ-QR  1055
HsATP8B1   LTSMILFFIPLGAYLQTV-GQDGEAPSD-YQSFAVTIASALVITVNFQIGLDTSYWTFVNAFSIFGSIALYFGIMFDFHSAGI-H-VLFPSAFQ  1131
HsATP8B2   YTSVLMFFIPYGVFADAT-RDDGTQLAD-YQSFAVTVATSLVIVVSVQIGLDTGYWTAINHFFIWGSLAVYFAILFAMHSNGL-F-DMFPNQFR  1071
HsATP10A   FQSLVCFSIPYLAYYDSN--------VD-LFTWGTPIVTIALLTFLLHLGIETKTWTWLNWITCGFSVLLFFT-VALIY-NAS-CATCYPP-SN  1260
AtALA2     FHAIIVFVITIHAYAYEK--------SE-MEELGMVALSGCIWLQAFVVAQETNSFTVLQHLSIWGNLVGFYA-INFLF-SA------IPS-SG   975
AtALA10    ISALAIFFLCKESLKHQL-FDPDGKTAG-REILGGTMYTCVVWVVNLQMALSISYFTWVQHIVIWGSIAFWYI-FLMIY-GAM-T-PSF-S-TD  1099
```

```
                                   TM10
CtDNF1     FYQA-APQVYQELTFWMCLIVTPALCLLPRLVVKCIQKQRFPYDVDIIREQAN--R---GDFAAAD------AAAV-----------AALGGPE  1362
CtDRS2     FFEV-IPRLFSNPSFWLQMPTLAILCLARDFAWKFSKRLWKPEAYHHVQEIQK--Y---NI---------------------------------  1300
ScDRS2     YYGV-VKHTYGSGVFWLTLIVLPIFALVRDFLWKYYKRMYEPETYHVIQEMQK--Y---NI---------------------------------  1246
ScDNF1     FFKA-AARIYGAPSFWAVFFVAVLFCLLPRFTYDSFQKFFYPTDVEIVREMWQ--H---GHFDHYP------PGYDPTDPNRPKVTKAGQHGEK  1446
ScDNF2     FYKG-AARVFAQPAYWAVLFVGVLFCLLPRFTIDCIRKIFYPKDIEIVREMWL--R---GDFDLYP------QGYDPTDPSRPRINEIRPLT-D  1488
BtATP8A2   MKGQ-ATMVLSSAHFWLGLFLVPTACLIEDVAWRAAKHTCKKTLLEEVQELEM--K---SR---------------------------------  1072
HsATP8A1   MSGE-AAMLFSSGVFWMGLLFIPVASLLLDVVYKVIKRTAFKTLVDEVQELEA--K---SQ---------------------------------  1092
HsATP8A2   MRGQ-ATMVLSSAHFWLGLFLVPTACLIEDVAWRAAKHTCKKTLLEEVQELET--K---SR---------------------------------  1112
HsATP11A   MYYV-FIQMLSSGPAWLAIVLLVTISLLPDVLKKVLCRQLWPTATERVQTKSQ--C---LSVE-------------------------------  1118
HsATP11C   MYFV-FAQMLSSVSTWLAIILLIFISLFPEILLIVLKNVRRRSARRNLSCRRASDS---LSAR-------------------------------  1114
HsATP8B1   FTGT-ASNALRQPYIWLTIILAVAVCLLPVVAIRFLSMTIWPSESDKIQKHRK--R---LK---------------------------------  1186
HsATP8B2   FVGN-AQNTLAQPTVWLTIVLTTVVCIMPVVAFRFLRLNLKPDLSDTVRYTQL--VRKKQK---------------------------------  1129
HsATP10A   PYWT-MQALLGDPVFYLTCLMTPVAALLPRLFFRSLQGRVFPTQLQLARQLTR--K---SPRRCSAPKETFAQGRLPKDS--------------  1334
AtALA2     MYTI-MFRLCSQPSYWITMFLIVGAGMGPIFALKYFRYTYRPSKINILQQAER--M---GGPIL-------TLG--------------------  1036
AtALA10    AYMVFLEALAPAPSYWLTTLFVMIFALIPYFVYKSVQMRFFPKYHQMIQWIRY--E---GHSN-------------------------------  1157

CtDNF1     RVEGESLG-SLSSSGKGSGRSKKSKHQQYASVDEDRRPIYPPSIATHNTRAQNGSD-------------GTT----------------------  1420
CtDRS2     ----------------------------------------------------------------------------------------------     -
ScDRS2     ----------------------------------------------------------------------------------------------     -
ScDNF1     IIEGIALSDNL----GGSNYSR-------DSVVTEEIPMTF-MH---------GED-------------GSPSGYQKQETW------------M  1494
ScDNF2     FKEPISLDTHF----DGVSHSQ-------ETIVTEEIPMSI-LN---------GEQ-------------GSRKGYRVSTTLERRDQLSPVTTTN  1548
BtATP8A2   ----------------------------------------------------------------------------------------------     -
HsATP8A1   ----------------------------------------------------------------------------------------------     -
HsATP8A2   ----------------------------------------------------------------------------------------------     -
HsATP11A   ----------------------------------------------------------------------------------------------     -
HsATP11C   ----------------------------------------------------------------------------------------------     -
HsATP8B1   ----------------------------------------------------------------------------------------------     -
HsATP8B2   ----------------------------------------------------------------------------------------------     -
HsATP10A   ---GT-----EHSSGR--------------TV-KTSVPLSQPS----------WHTQQPVCSLEASGEPS------------------------  1371
AtALA2     ----N-----IETQPR--------------TIEKDLSPISITQ----------PKNRSPVY---------------------------------  1064
AtALA10    -------------------------------------------------------------------DPE------------------------  1160

CtDNF1     -------------------YIMQSRTS------------TELQQEMPFDRDREEETPAVR-PSIERTRPSYDRIRR--SIDRVRPSFEASNDFT  1480
CtDRS2     ----------------------------------------------------QDYRP---------RME---QFQK--AIRKVR----------  1318
ScDRS2     ----------------------------------------------------SDSRP---------HVQ---QFQN--AIRKVR----------  1264
ScDNF1     TSPKETQDLLQSPQFQQAQTFG-------------------------------------R--------GPSTNVRS--SLDRTREQMIATNQLD  1541
ScDNF2     NLPRRSMASAR---------------------------------------------------------G--NKLRT--SLDRTREEMLANHQLD  1581
BtATP8A2   ----------------------------------------------------VMGRAMLR-DSNGKRMNERDRLLK--RLSRKTP---------  1102
HsATP8A1   ----------------------------------------------------DPGAVVL-----GKSLTERAQLLK--NVFKKNH---------  1118
HsATP8A2   ----------------------------------------------------VLGKAVLR-DSNGKRLNERDRLIK--RLGRKTP---------  1142
HsATP11A   ---------------------------------------------------------------------------------QSTI---------  1122
HsATP11C   ---------------------------------------------------------------------------------PSVR---------  1118
HsATP8B1   ----------------------------------------------------AE-----------------EQWQRRQQVFR------------  1199
HsATP8B2   ----------------------------------------------------AQ-----------------HRCMR--RVGR------------  1140
HsATP10A   --------------------------TVDMSMPVREHTLLEGLSAP-APMSSAPGEAVLR-SPGGCPEESKVRA-A--STGRVTP---------  1425
AtALA2     --------------------------------------------------EP-----LLSDSPN-ATR-------R--SFGPGTP---------  1084
AtALA10    --------------------------FV------------EMVRQR-SIRPTTVGYTARR-AA--------------------------------  1183

CtDNF1     SAARL------------SRIESTHSSL--GHTYSHQRESYAG--ESSGAQQGQEP--GQRRFNLATVRKRGLSAFSKKSIDTT--EGE------  1548
CtDRS2     ------------------QVQRM--RKQRGYAFSMA--DE------------SQTR--VLQAY------------------DTT--RHR------  1351
ScDRS2     ------------------QVQRM--KKQRGFAFSQA--EEG-----------GQEK--IVRMY------------------DTT--QKR------  1298
ScDNF1     NRYSV------------ERARTSL------------DL--------PGVTN---------A-----------ASL-------I--GTQ------  1568
ScDNF2     TRYSV------------ERARASL------------DL--------PGINH---------A-----------ETL-------L--SQR------  1608
BtATP8A2   --PT-L-----------FRGSSLQQSMPHGYAFSQE--EHGA--V------TQEE--IVRAY------------------DTT--KQK------  1144
HsATP8A1   --VN-L-----------YRSESLQQNLLHGYAFSQD--ENGI--V------SQSE--VIRAY------------------DTT--KQR------  1160
HsATP8A2   --PT-L-----------FRGSSLQQGVPHGYAFSQE--EHGA--V------SQEE--VIRAY------------------DTT--KKK------  1184
HsATP11A   --FML-S-----------QTSSS-------LSF-------------------------------------------------------------  1134
HsATP11C   --PLL-L-----------RTFSD-------ESN---------------VL--------------------------------------------  1132
HsATP8B1   ------------------RGVST---RRSAYAFSHQ--RGYADLI------SSGRSIRKKRS------------------PLDAIVADGTAE--  1244
HsATP8B2   ----------------------T-GSRRSGYAFSHQ--EGFGELI------MSGKNMRLSSL------------------ALSSFTTRSSSSWI  1185
HsATP10A   --LSSLFSLPTFSLLNWISSWSLVSRLGSVLQFSRT--EQLADGQ-----AGRG-------L------------------PVQ--PHS------  1477
AtALA2     --FE-F-----------FQSQSR-LSSSSGYTRNCK--D-------------------------------------------------------  1106
AtALA10    ---------------------SV--------------------------------------------------------------RRS------  1188

CtDNF1     ----PPREP-------------------------------------------PM------------------  1555
CtDRS2     ----GR-YGEMAS--------------------------------------SRT-IGI------AQ------  1367
ScDRS2     ----GK-YGELQDASANPFNDNNGLGSNDFESAEPFIENPFADGNQNSNRFSSSRDDI------SF-DI---  1355
ScDNF1     ----Q-N-N---------------------------------------------------------------  1571
ScDNF2     ----S-RDR---------------------------------------------------------------  1612
BtATP8A2   ----SR-KK---------------------------------------------------------------  1148
HsATP8A1   ----PD-EW---------------------------------------------------------------  1164
HsATP8A2   ----SR-KK---------------------------------------------------------------  1188
HsATP11A   ------------------------------------------------------------------------     -
HsATP11C   ------------------------------------------------------------------------     -
HsATP8B1   --YR-R-TGD------------------------------------------------------------S-  1251
HsATP8B2   ESLRRK-KSDSAS--------------------------------------SPS-GGA------DK-PLKG-  1209
HsATP10A   ----GR-SG--------------------------------------------L-QGPDHRLLIGASSRRSQ  1499
AtALA2     -----------------------------------------------------------------------N  1107
AtALA10    ----AR-FHDQIY--------------------------------------KDL-VGV--------------  1202
```

Fig S2

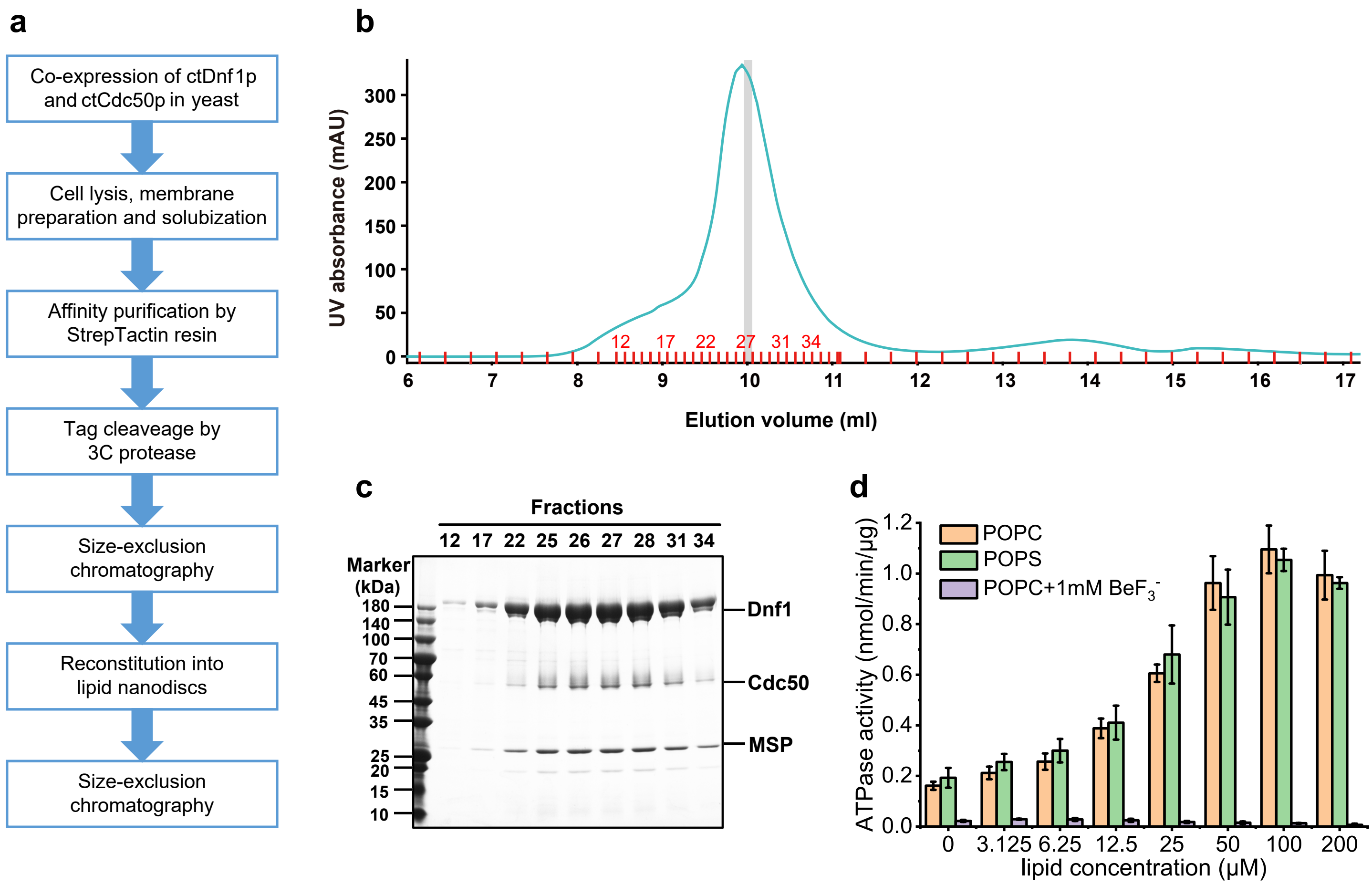

a
Co-expression of ctDnf1p and ctCdc50p in yeast
Cell lysis, membrane preparation and solubization
Affinity purification by StrepTactin resin
Tag cleaveage by 3C protease
Size-exclusion chromatography
Reconstitution into lipid nanodiscs
Size-exclusion chromatography
b
UV absorbance (mAU)
Elution volume (ml)
12 17 22 27 31 34
c
Fractions
12 17 22 25 26 27 28 31 34
Marker (kDa)
180 140 100 70 60 45 35 25 20 15 10
Dnf1
Cdc50
MSP
d
ATPase activity (nmol/min/μg)
POPC
POPS
POPC+1mM BeF3-
0 3.125 6.25 12.5 25 50 100 200
lipid concentration (μM)

Fig S3

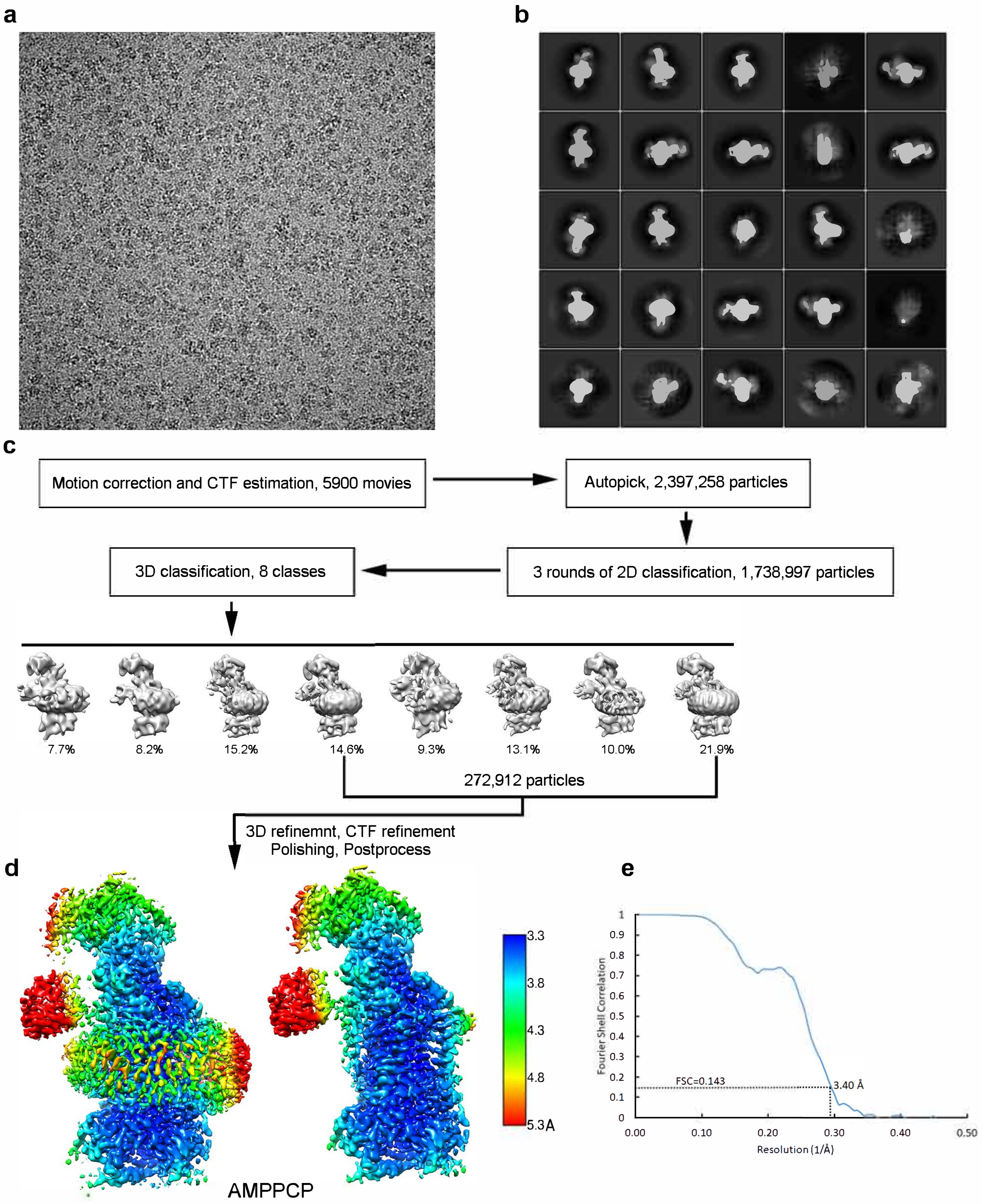
a
b
c
Motion correction and CTF estimation, 5900 movies
Autopick, 2,397,258 particles
3 rounds of 2D classification, 1,738,997 particles
3D classification, 8 classes
7.7%
8.2%
15.2%
14.6%
9.3%
13.1%
10.0%
21.9%
272,912 particles
3D refinemnt, CTF refinement
Polishing, Postprocess
d
e
3.3
3.8
4.3
4.8
5.3Å
AMPPCP
Fourier Shell Correlation
Resolution (1/Å)
FSC=0.143
3.40 Å
0.00
0.10
0.20
0.30
0.40
0.50

Fig. S4

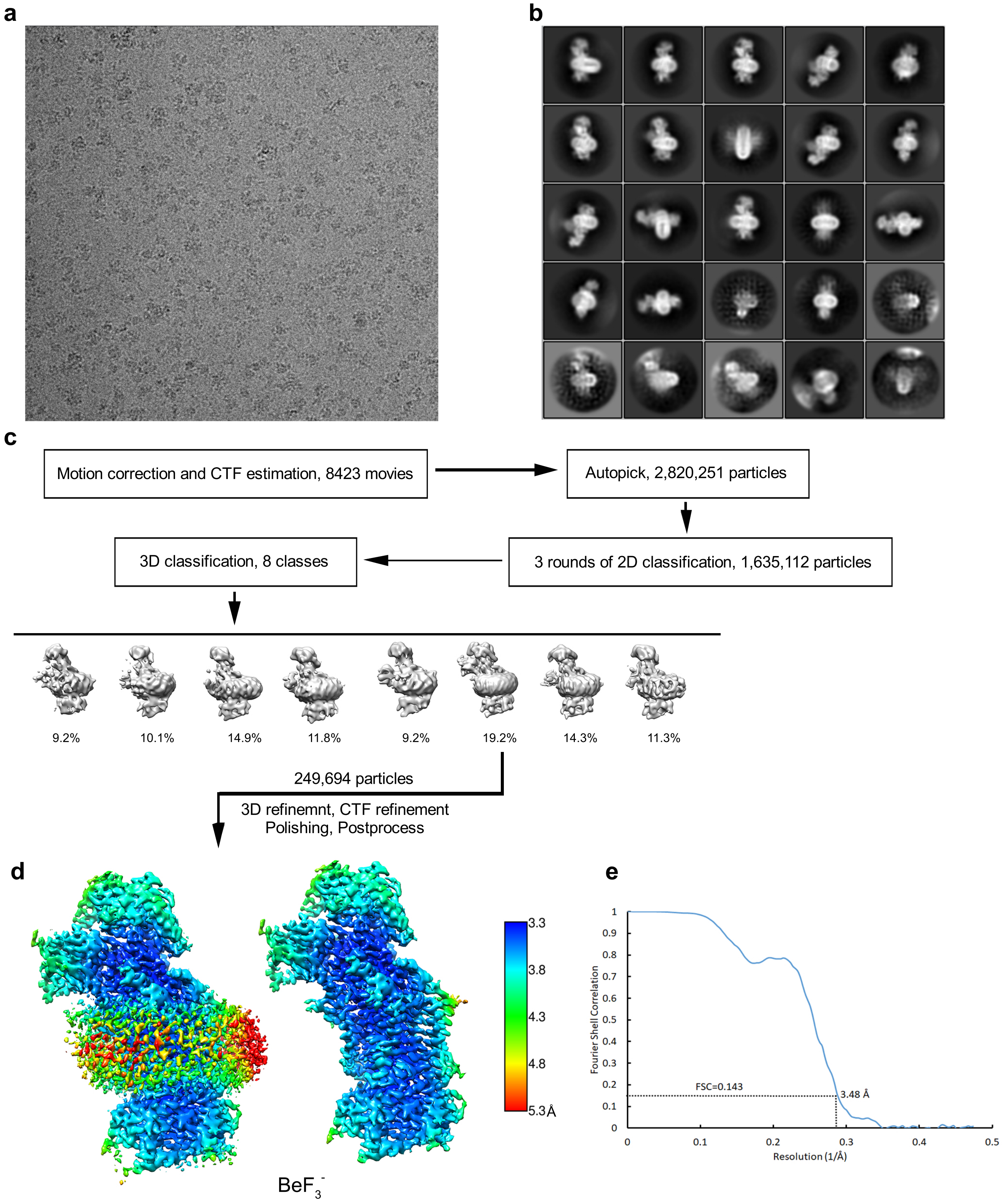
a
b
c
Motion correction and CTF estimation, 8423 movies
Autopick, 2,820,251 particles
3 rounds of 2D classification, 1,635,112 particles
3D classification, 8 classes
9.2%
10.1%
14.9%
11.8%
9.2%
19.2%
14.3%
11.3%
249,694 particles
3D refinemnt, CTF refinement
Polishing, Postprocess
d
3.3
3.8
4.3
4.8
5.3Å
BeF3-
e
Fourier Shell Correlation
Resolution (1/Å)
FSC=0.143
3.48 Å
0
0.1
0.2
0.3
0.4
0.5
0.6
0.7
0.8
0.9
1

Fig. S5

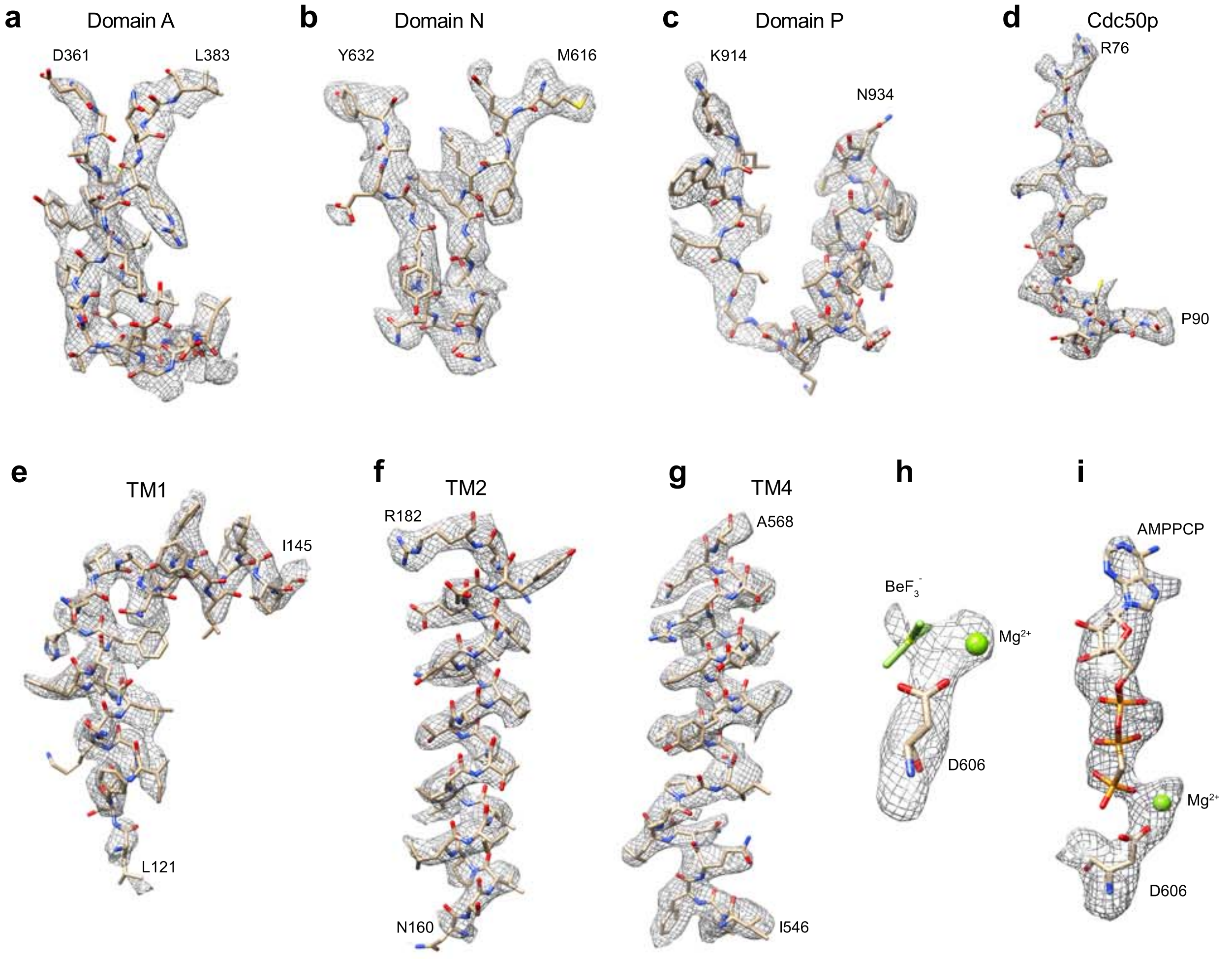

a
Domain A
D361
L383
b
Domain N
Y632
M616
c
Domain P
K914
N934
d
Cdc50p
R76
P90
e
TM1
I145
L121
f
TM2
R182
N160
g
TM4
A568
I546
h
BeF3-
Mg2+
D606
i
AMPPCP
Mg2+
D606

Fig. S6

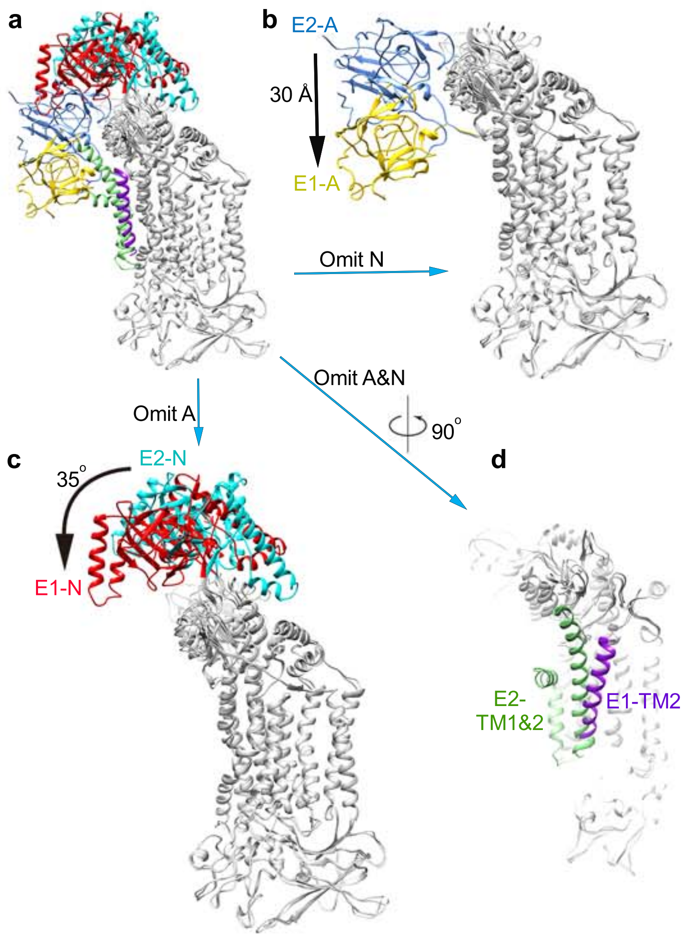
a
b
E2-A
30 Å
E1-A
Omit N
Omit A&N
90°
Omit A
c
E2-N
35°
E1-N
d
E2-
TM1&2
E1-TM2

Fig. S7

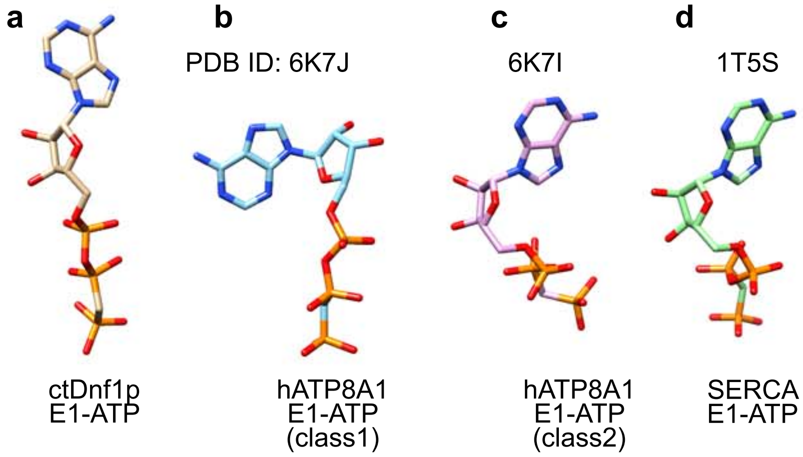
a
b
PDB ID: 6K7J
c
6K7I
d
1T5S
ctDnf1p
E1-ATP
hATP8A1
E1-ATP
(class1)
hATP8A1
E1-ATP
(class2)
SERCA
E1-ATP

Fig. S8

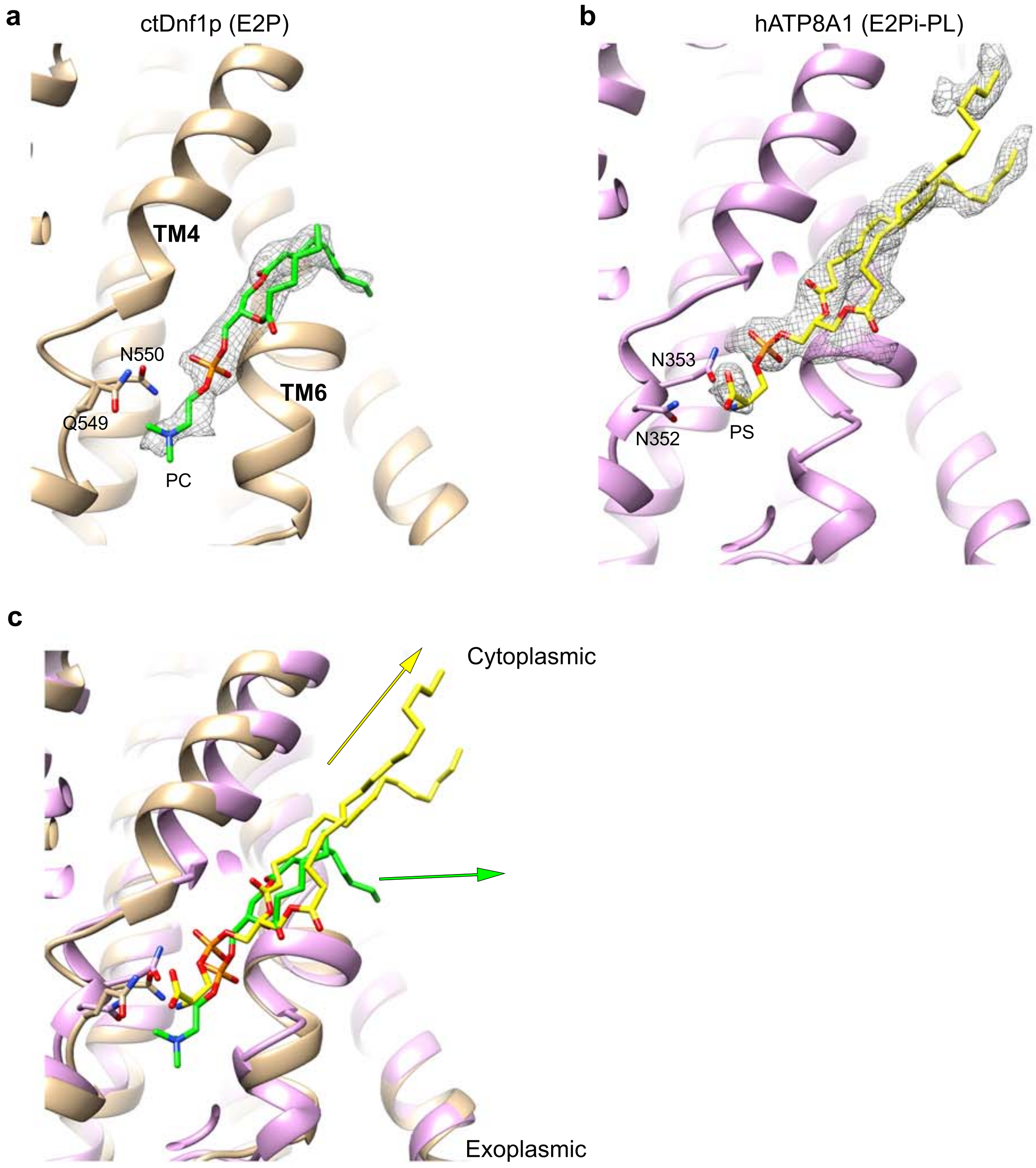
a
ctDnf1p (E2P)
TM4
N550
Q549
TM6
PC
b
hATP8A1 (E2Pi-PL)
N353
N352
PS
c
Cytoplasmic
Exoplasmic

Fig. S9

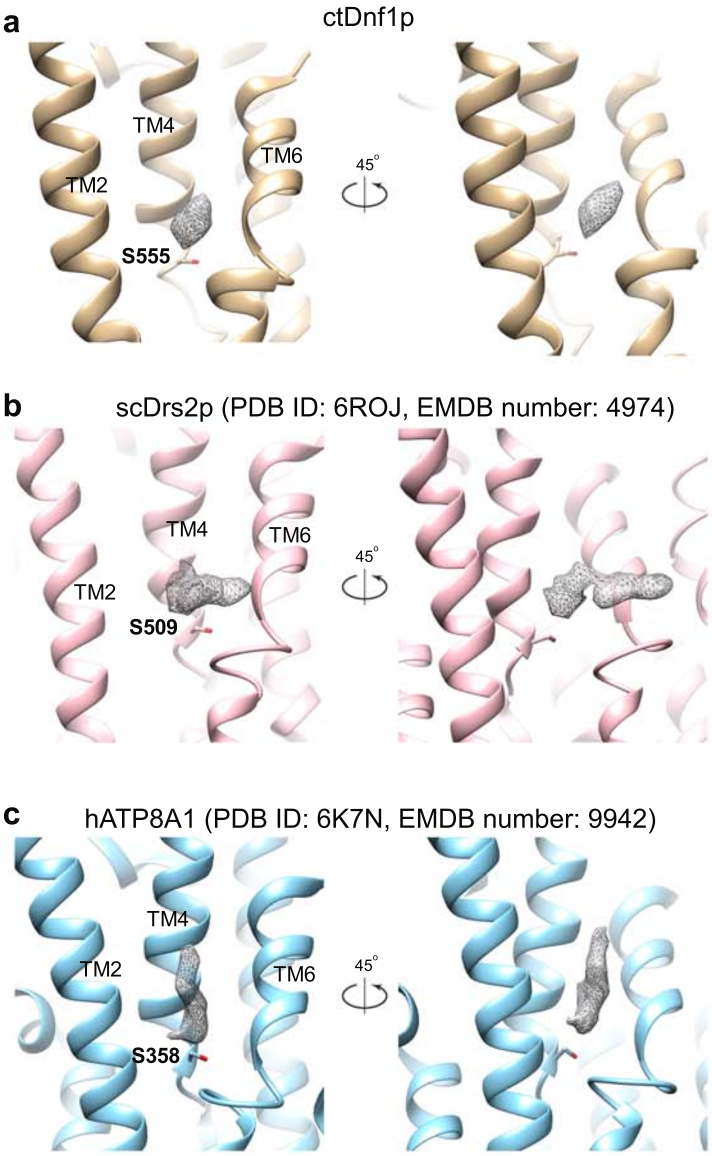
a
ctDnf1p
TM4
TM6
TM2
45°
S555
b
scDrs2p (PDB ID: 6ROJ, EMDB number: 4974)
TM4
TM6
TM2
45°
S509
c
hATP8A1 (PDB ID: 6K7N, EMDB number: 9942)
TM4
TM2
TM6
45°
S358

Fig. S10

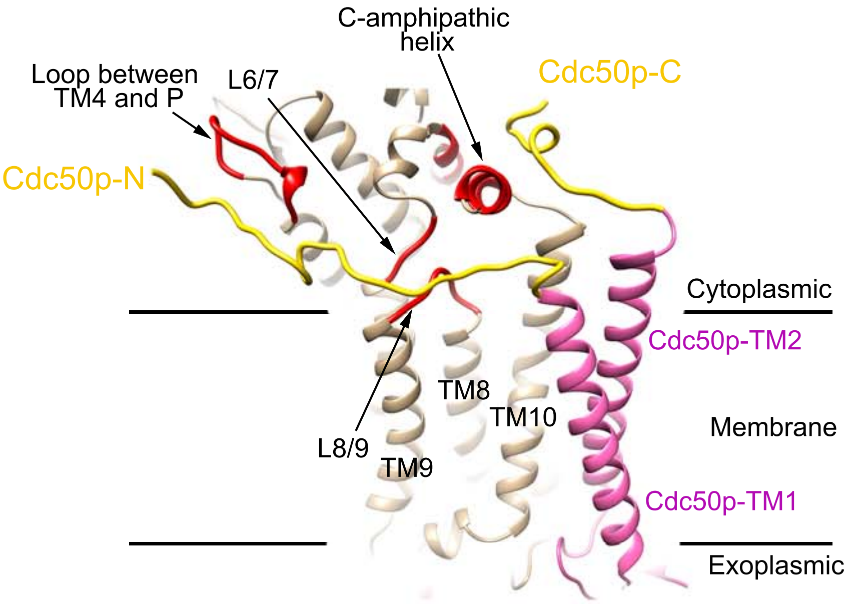
C-amphipathic helix
Loop between TM4 and P
L6/7
Cdc50p-C
Cdc50p-N
Cytoplasmic
Cdc50p-TM2
TM8
TM10
Membrane
L8/9
TM9
Cdc50p-TM1
Exoplasmic

Fig. S11

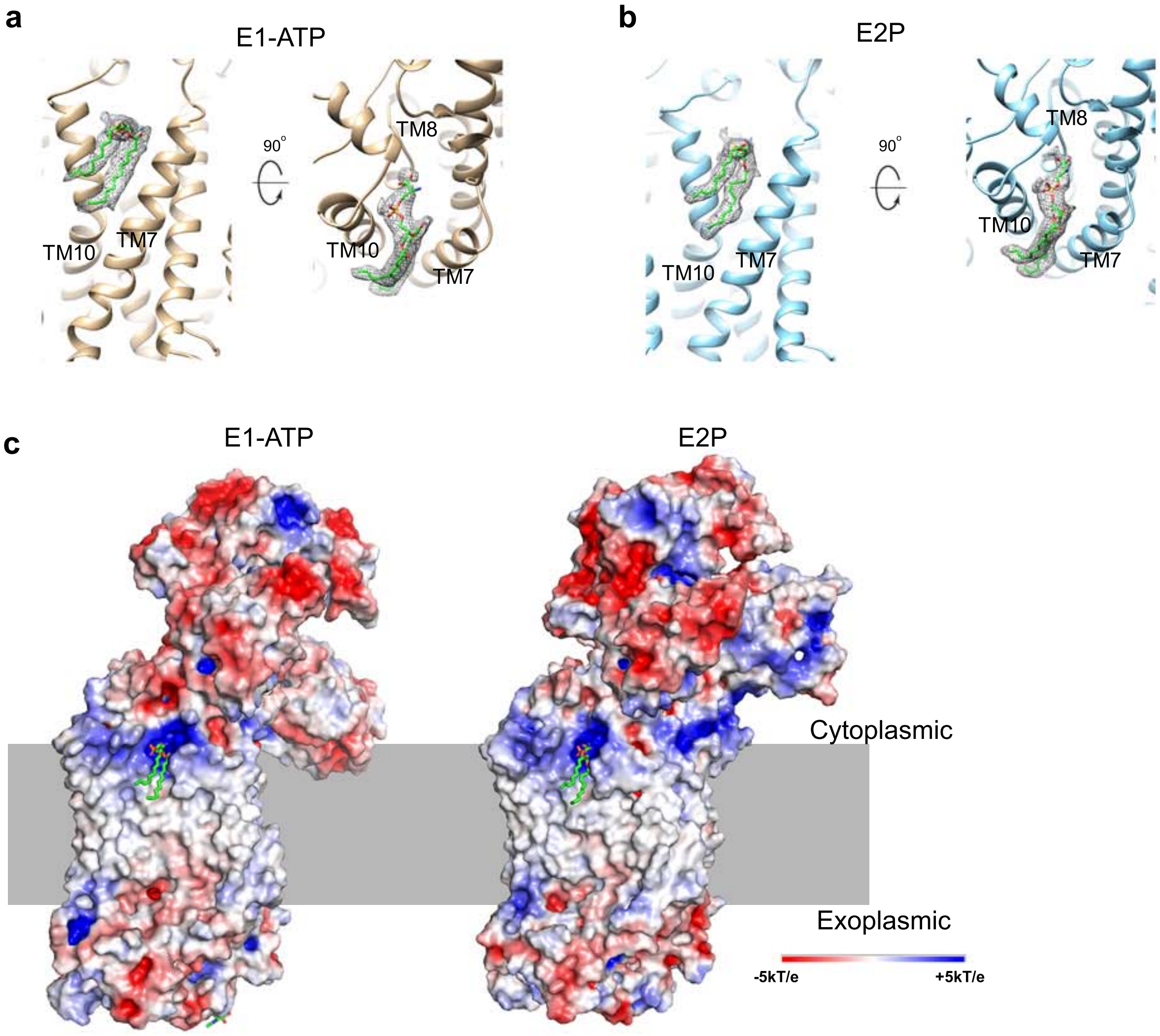

a
E1-ATP
90°
TM8
TM10
TM7
TM10
TM7
b
E2P
90°
TM8
TM10
TM7
TM10
TM7
c
E1-ATP
E2P
Cytoplasmic
Exoplasmic
-5kT/e
+5kT/e

Fig. S12

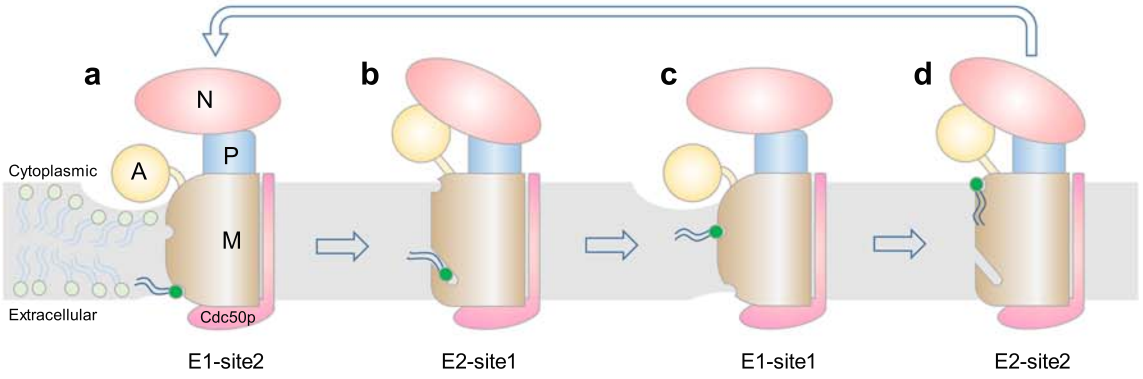

a
b
c
d
N
P
A
M
Cytoplasmic
Extracellular
Cdc50p
E1-site2
E2-site1
E1-site1
E2-site2

Fig. S13

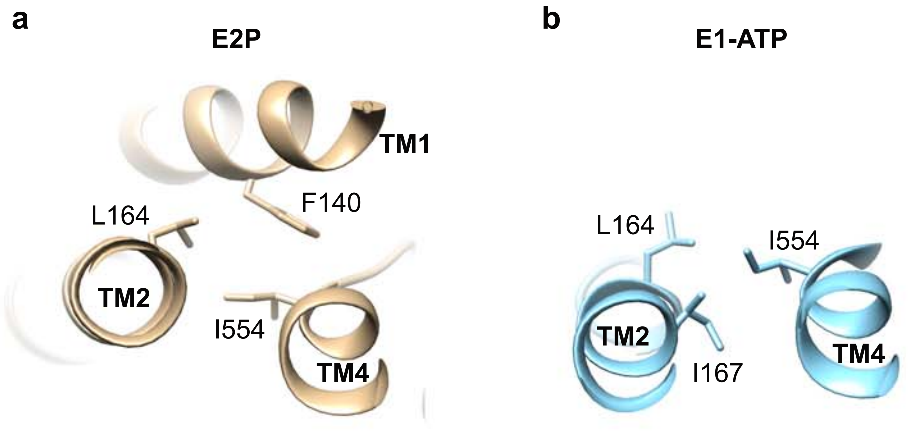

Supplement: Supplementary file 1 — Supplementary material 1 (PDF 7282 kb) [file 13238_2020_712_MOESM1_ESM.pdf]
